# HCV prevalence can predict HIV epidemic potential among people who inject drugs: mathematical modeling analysis

**DOI:** 10.1186/s12889-016-3887-y

**Published:** 2016-12-03

**Authors:** Vajiheh Akbarzadeh, Ghina R. Mumtaz, Susanne F. Awad, Helen A. Weiss, Laith J. Abu-Raddad

**Affiliations:** 1 Infectious Disease Epidemiology Group, Weill Cornell Medical College – Qatar, Cornell University, Doha, Qatar; 2 Department of Healthcare Policy and Research, Weill Cornell Medical College, Cornell University, New York, USA; 3 Department of Infectious Disease Epidemiology, Faculty of Epidemiology and Population Health, London School of Hygiene and Tropical Medicine, Keppel Street, London, WC1E 7HT UK; 4 Department of Infectious Disease Epidemiology, MRC Tropical Epidemiology Group, Faculty of Epidemiology and Population Health, London School of Hygiene and Tropical Medicine, London, UK; 5 College of Public Health, Hamad bin Khalifa University, Doha, Qatar

**Keywords:** HIV, Hepatitis C virus, People who inject drugs, Mathematical modeling, Prediction

## Abstract

**Background:**

Hepatitis C virus (HCV) and HIV are both transmitted through percutaneous exposures among people who inject drugs (PWID). Ecological analyses on global epidemiological data have identified a positive association between HCV and HIV prevalence among PWID. Our objective was to demonstrate how HCV prevalence can be used to predict HIV epidemic potential among PWID.

**Methods:**

Two population-level models were constructed to simulate the evolution of HCV and HIV epidemics among PWID. The models described HCV and HIV parenteral transmission, and were solved both deterministically and stochastically.

**Results:**

The modeling results provided a good fit to the epidemiological data describing the ecological HCV and HIV association among PWID. HCV was estimated to be eight times more transmissible per shared injection than HIV. A threshold HCV prevalence of 29.0% (95% uncertainty interval (UI): 20.7-39.8) and 46.5% (95% UI: 37.6-56.6) were identified for a sustainable HIV epidemic (HIV prevalence >1%) and concentrated HIV epidemic (HIV prevalence >5%), respectively. The association between HCV and HIV was further described with six dynamical regimes depicting the overlapping epidemiology of the two infections, and was quantified using defined and estimated measures of association. Modeling predictions across a wide range of HCV prevalence indicated overall acceptable precision in predicting HIV prevalence at endemic equilibrium. Modeling predictions were found to be robust with respect to stochasticity and behavioral and biological parameter uncertainty. In an illustrative application of the methodology, the modeling predictions of endemic HIV prevalence in Iran agreed with the scale and time course of the HIV epidemic in this country.

**Conclusions:**

Our results show that HCV prevalence can be used as a proxy biomarker of HIV epidemic potential among PWID, and that the scale and evolution of HIV epidemic expansion can be predicted with sufficient precision to inform HIV policy, programming, and resource allocation.

**Electronic supplementary material:**

The online version of this article (doi:10.1186/s12889-016-3887-y) contains supplementary material, which is available to authorized users.

## Background

Prioritization of populations and settings for HIV prevention interventions is critical to increase the cost-effectiveness of programs [1]. This is particularly the case in resource-limited settings such as most of the Middle East and North Africa (MENA) where HIV surveillance among most-at-risk populations, including people who inject drugs (PWID), remains deficient [2]. In this region, emerging and sometimes rapidly rising HIV epidemics have been recently documented among PWID [3]. Identification of settings with high HIV epidemic potential among PWID would help in prioritization and resource allocation for prevention interventions before HIV prevalence reaches high endemic levels. In this work, we provide the theoretical foundation, and describe an application in MENA, for an innovative approach to identify PWID populations at high risk of future HIV epidemic expansion. The concept is to use prevalence data on hepatitis C virus (HCV) to predict HIV epidemic potential.

Both HCV and HIV are transmitted through percutaneous exposures, and among PWID, sharing of non-sterile injecting equipment is the main mode of transmission [4]. However, HCV is more transmissible than HIV [5], has a higher prevalence, and is hyperendemic in most PWID populations [6, 7]. Globally, 63% of PWID are HCV infected [6, 7] while only 19% are HIV infected [6]. A recent meta-analysis identified that 82% of HIV-infected PWID are co-infected with HCV [8]. At the individual level, with HCV being most often transmitted before HIV along the same route of transmission, it could be used as a marker of the risk of exposure to HIV.

Few studies have investigated the association between HCV and HIV among PWID [9–13]. Ecological analyses on global [10] and MENA [9] epidemiological data have identified a positive association between the two infections. The association was found to be most robust when both infections are at endemic equilibrium [9], and is characterized by a threshold effect whereby HIV prevalence is likely to be negligible below a certain HCV prevalence of about 30% [10]. Two mathematical modeling studies reproduced the epidemiological epidemic dynamics [11, 13]. They projected the presence of an HCV threshold effect for a sustainable HIV epidemic; but the value of this HCV threshold was found to be highly sensitive to a number of behavioral parameters such as heterogeneity in risk, level of mixing, and duration of injecting [11, 13]. The models were also able to reproduce, above the threshold, the diversity of HCV and HIV epidemics occurring in different settings [11, 13]. These models however, with the uncertainty in behavioral parameters, questioned the utility of HCV prevalence in predicting HIV epidemic scale.

In this study, we re-examine the HCV-HIV association among PWID using a modeling approach that accommodates stochasticity and a complex injecting contact structure. We estimate the HCV thresholds for HIV epidemic expansion, and assess the extent to which this threshold is affected by variations in behavioral and HCV/HIV biological parameters. We also examine new aspects in the HCV-HIV association that include 1) estimating the HCV to HIV infectiousness ratio, 2) identifying the different dynamical regimes in the overlapping HCV-HIV epidemiology, and 3) developing and estimating summary measures that quantify the association between the two infections and that can be used, beyond modeling, for predictions of HIV epidemic potential among PWID. We further quantify the margins in HCV predictability of HIV epidemic scale across a wide range of HCV prevalence, through the conduct of uncertainty analyses. Finally, an application is provided for one MENA country where the HCV-HIV association is used to predict the scale and evolution of the HIV epidemic.

## Methods

### HCV and HIV models structure

**Fig. 1:**
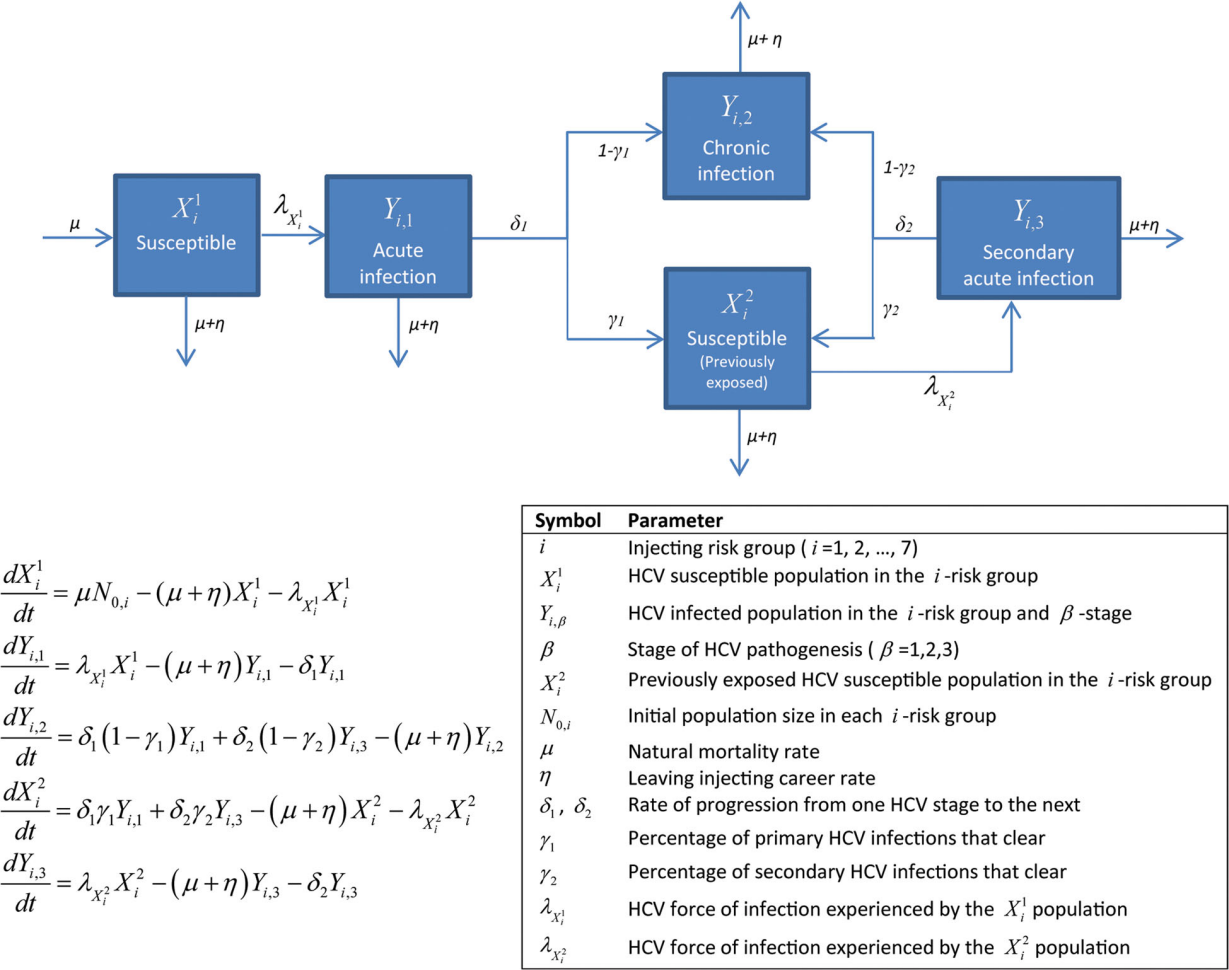
HCV mathematical model description, equations, and parameter definitions. The details pertaining to the force of infection can be found in Additional file 1

**Fig. 2:**
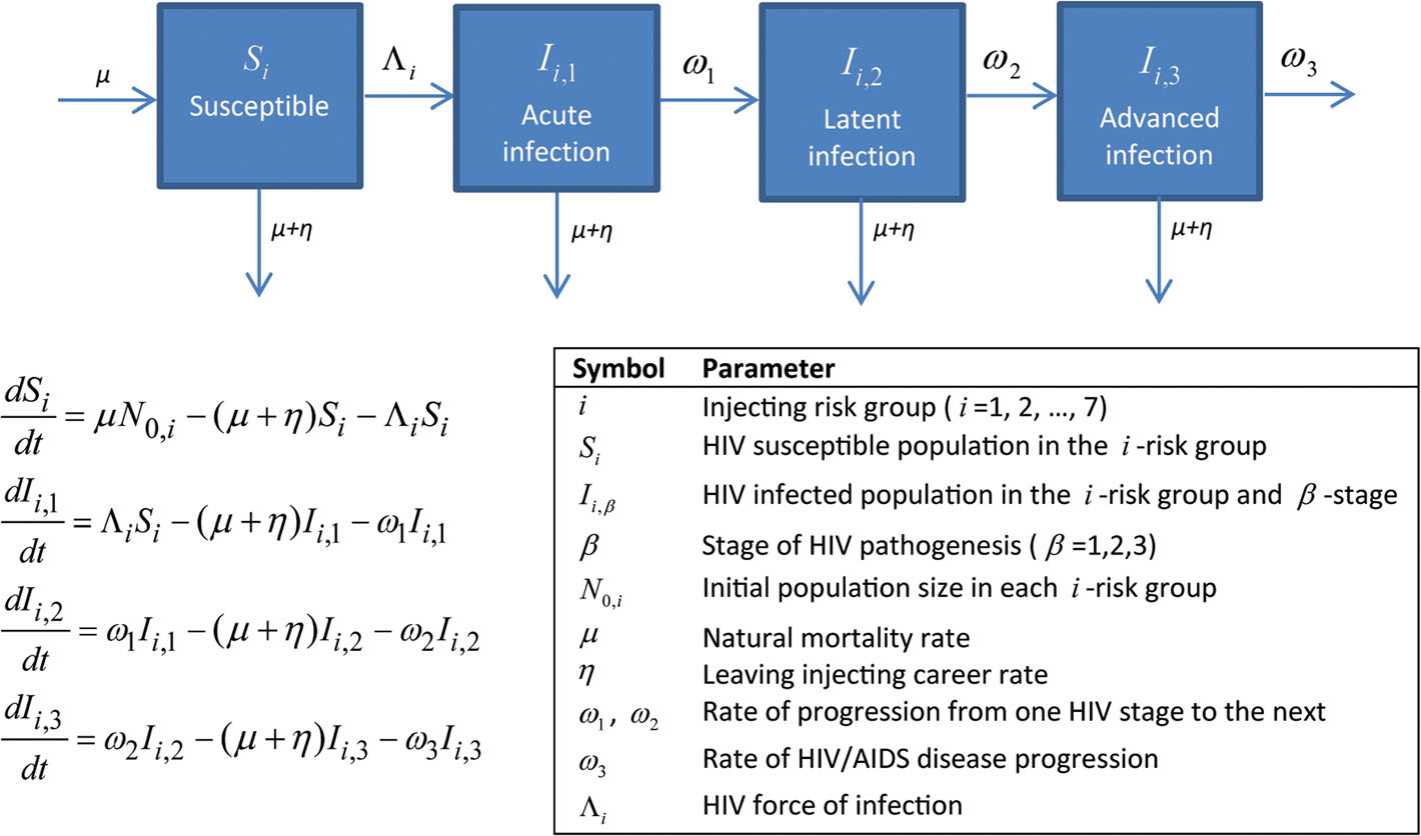
HIV mathematical model description, equations, and parameter definitions. The details pertaining to the force of infection can be found in Additional file 1

Two population-level compartmental models were constructed to simulate the evolution of HCV and HIV epidemics among PWID (Figs. 1 and 2, and Additional file 1). The models describe HCV/HIV parenteral transmission through sharing of non-sterile needles/syringes, and were solved both deterministically and stochastically. The deterministic versions of the models were expressed each through a system of coupled nonlinear differential equations, and stratified the PWID population into compartments according to HCV/HIV status, stage of HCV/HIV infection, and level of injecting risk behavior. The stochastic versions used the same transition rates in the deterministic systems to generate the stochastic processes. HIV progression in the HIV model was divided into three stages: acute, latent, and advanced; while progression in the HCV model was divided into stages of acute (primary infection), chronic, and secondary acute. The latter denotes the acute phase following HCV reinfection, in the event the primary infection was cleared.

Both the HCV and HIV models used the same injecting behavior structure and parameter values. To accommodate heterogeneity in injecting risk behavior, we stratified the PWID population into seven risk groups with increasing level of injecting risk behavior. We assumed that individuals become PWID at a constant rate and remain in the same risk group until the end of their injecting career or death. We also assumed that the distribution of the PWID population across the seven risk groups follows a gamma distribution, as motivated by previous theoretical and applied mathematical modeling work [14–19] (Eq. 1 and Additional file 1).

1 $$ p(i)=\frac{1}{b^a\varGamma (a)}{i}^{a-1}{e}^{\frac{-i}{b}} $$

Here *a* is the shape parameter and *b* is the scale parameter in the gamma distribution. With such a distribution, the majority of the PWID population belongs to relatively lower risk groups while a small fraction belongs to the higher risk groups. PWID of different risk groups interact according to a mixing matrix with a continuous spectrum between assortative (choosing injecting partners from within their risk group) and proportionate (choosing partners with no preferential bias based on the type of risk group) mixing.

The level of risk behavior was modeled by the effective partnership change rate. While expressed in units of injecting partners per year, the effective partnership change rate is a complex summary measure of the overall risk of exposure to HCV/HIV infections. It captures effectively different factors that reflect the nature of injecting risk behavior and networks, but are difficult to quantify, such as clustering within networks, concurrency, and variability in risk behavior [20–24]. Accordingly, the effective partnership change rate reflects the distribution and strength of the risk of exposure to HCV/HIV infection. Motivated by previous mathematical modeling work [25–29], the distribution of the level of injecting risk behavior, that is of the effective partnership change rate, across the seven risk groups was defined through the following power law function where the level of risk behavior grows larger and larger with the risk group number (Eq. 2 and Additional file 1):

2 $$ {\rho}_{P_i} = C{i}^{\alpha } $$

where *α* is the exponent in the power-law function and *C* is an overall constant. The different HCV/HIV epidemic scales were generated by changing the value of the average effective partnership change rate in the PWID population. Further details on model structure can be found in Additional file 1.

### Data sources and model fitting

The model parameters were derived using recent empirical HCV/HIV natural history and epidemiology data, as well as through model fitting for some of the parameters. All HCV/HIV biological and behavioral parameter values and their references are summarized in Additional file 2. Further justification for the parameter values are provided in Additional file 1, Section 4.

HCV and HIV model predictions were fitted to global epidemiological HCV and HIV prevalence data among PWID [10]. These data were identified in an earlier systematic review of literature and included 863 paired HCV-HIV data points among PWID from 343 different geographical areas in 61 countries [10]. The paired HCV-HIV prevalence data were then fitted to a statistical segmented linear regression model, indicating a positive ecological association between HCV and HIV prevalence [10]. Our modeling predictions of endemic HCV and HIV prevalence at various levels of injecting risk behavior were fitted to this ecological statistical association describing the epidemiological global HCV-HIV data [10]. The main purpose of the fitting was to determine the best-fit value for the HCV/HIV infectiousness ratio (that is the ratio of the HCV transmission probability per shared injection to that of HIV), a biological parameter and not a population-specific parameter. This fitting insured that our modeling predictions describe the actual HCV-HIV association observed empirically among PWID. The fitting was made in the range of HCV prevalence of 40–60%, where there is the highest volume of epidemiological data [10]. We used a nonlinear least-square fitting method incorporating the Nelder-Mead simplex algorithm as described in Lagarias et al. to find the best fit [30]. The method, as well as most of our modeling analyses, were implemented in MATLAB [31, 32].

In addition to the infectiousness ratio of HCV to HIV, three other measures were derived through the optimum fit to the global epidemiological data: The scale and shape parameters of the gamma distribution of the population across injecting risk groups, and the exponent parameter of the power law distribution of the level of injecting risk behavior across risk groups.

### Plan of analysis

#### HCV thresholds for HIV epidemic expansion

##### Derivation

After fitting to global epidemiological data, we used the best-fit parameters in applying the HCV and HIV models. As a first step, we examined the association between the prevalence of the two infections in broad epidemic scales by plotting, at endemic equilibrium, HIV prevalence as a function of HCV prevalence. This was done by varying the average injecting risk behavior parameter (effective partnership change rate) and generating endemic HCV and HIV prevalence for each value of average injecting risk behavior, using the deterministic versions of the models. The endemic HCV prevalence at which the corresponding endemic HIV prevalence became greater than 1% was identified as the HCV threshold for sustainable HIV epidemic, and the endemic HCV prevalence that corresponded to an endemic HIV prevalence of 5% was identified as the threshold for concentrated HIV epidemic.

##### Sensitivity analysis

We conducted extensive univariate sensitivity analyses to explore the HCV-HIV association at broad ranges of changes in each model parameter, including not only plausible but also extreme values that are not even seen empirically. We examined the sensitivity of our modeling predictions of the HCV thresholds, for both sustainable and concentrated HIV epidemics, to variations in 1) the infectiousness ratio of HCV to HIV, 2) several injecting risk behavior parameters including: the degree of assortative mixing, the scale and shape parameters of the gamma distribution of the population across injecting risk groups, the exponent parameter of the power law distribution of the level of injecting risk behavior, and the duration of injecting and 3) scale-up of antiretroviral therapy (ART) among those eligible for treatment. We assumed that all infected PWID in the advanced HIV stage and half of those in the latent HIV stage would be eligible for ART treatment, which corresponds roughly to a CD4 cell count criterion for treatment of 500 cells/μl [33]. We assumed that the efficacy of ART in reducing HIV transmission among PWID is 100%, based on a clinical trial of treatment for prevention and other observational data [34, 35]. We also assumed that, by slowing disease progression, 100% coverage among those eligible for ART would double the average duration from onset of infection to death among the total HIV infected population. Wide ranges of values for the parameters of the sensitivity analyses were chosen to produce a broad range of epidemics.

### Uncertainty analyses

Two separate multivariate uncertainty analyses were conducted to specify ranges of uncertainty in the predicted HCV thresholds, for both sustainable and concentrated HIV epidemics, with respect to 1) biological parameters and 2) behavioral parameters. The biological parameters that were varied included: the probabilities of HCV and HIV transmission per shared injection in each infection stage, the duration of each HCV and HIV stage, and the proportions of virus clearance for HCV primary infection and HCV reinfection. The behavioral parameters that were varied included: the duration of injecting, the degree of assortative mixing, the scale and shape parameters of the gamma distribution of the population across risk groups, the exponent parameter of the power law distribution of the level of risk behavior, and the frequency of sharing acts per partnership. These were the same set of parameters that were varied in all subsequent behavioral uncertainty analyses (as discussed below).

The parameters of the uncertainty analyses were varied within 20% of their point estimates (Additional file 2). We implemented 5,000 runs of the deterministic HCV and HIV models using Monte Carlo sampling from uniform probability distributions for the uncertainty in these parameters. Estimates for the mean values and associated 95% uncertainty intervals (UI) for the predicted HCV thresholds were determined by fitting a log-normal distribution to the range of values as described elsewhere [36].

#### Overlapping epidemiology of HCV and HIV infections

We quantified the epidemiological association between HCV and HIV among PWID using the risk ratio (*RR*_*HCV*/*HIV*_) and odds ratio (*OR*_*HCV*/*HIV*_) of HCV prevalence to HIV prevalence. The two measures were defined as follows:

3 $$ R{R}_{HCV/HIV}=\frac{P_{HCV}-{P}_{threshold}}{P_{HIV}} $$

4 $$ O{R}_{HCV/HIV}=\left(\frac{P_{HCV}-{P}_{threshold}}{1-\left({P}_{HCV}-{P}_{threshold}\right)}\right)/\left(\frac{P_{HIV}}{1-{P}_{HIV}}\right) $$

where *P*_*HCV*_ is HCV prevalence, *P*_*threshold*_ is the minimum HCV prevalence for a sustainable HIV epidemic, and *P*_*HIV*_ is HIV prevalence.

HCV and HIV prevalence at endemic equilibrium, *RR*_*HCV*/*HIV*_, and *OR*_*HCV*/*HIV*_ were examined as a function of the average injecting risk behavior, that is the effective partnership change rate, to qualitatively and quantitatively describe the overlapping dynamics of the two infections at variable epidemic scales.

#### Effect of behavioral uncertainty on HCV-based predictions of HIV epidemic scale

Our overarching aim is to use the above observed association between HCV and HIV infections to predict the future size of HIV epidemics using HCV prevalence. We assessed the precision of HCV prevalence in predicting HIV prevalence at endemic equilibrium by quantifying, across the whole spectrum of HCV prevalence, the effect of behavioral uncertainty on our modeling prediction of HIV prevalence. For each value of HCV prevalence, increasing in increments of 4%, we implemented 50 runs of the deterministic model using the uncertainty analysis methods described above. The new set of parameter values was used to refit the model to the specific HCV prevalence. We then compared, at each HCV prevalence level, the difference between the baseline prediction of HIV prevalence and the 50 predictions of HIV prevalence including the behavioral uncertainty.

#### Application to Iran

##### HIV epidemic size prediction

We applied the concept of using HCV prevalence to predict HIV epidemic scale in Iran as an illustrative example. We applied the deterministic version of the HCV model, and varied the average injecting risk behavior until the model generated the observed HCV prevalence in Iran. This specific value of the average injecting risk behavior was then used in applying the stochastic HIV model and predicting the time course of the HIV epidemic in this country.

The predicted time course of the HIV epidemic was compared to the observed HIV prevalence levels in the two conducted nationally-representative surveillance surveys among PWID in Iran [37, 38]. In addition to these two quality national data points, there are close to 100 HIV point prevalence measures over time among PWID in Iran [3]. These data show a clear trend for the HIV PWID epidemic which started its emergence in the late 1990s, reached a peak around the year 2005, then stabilized over the last decade or so at a national prevalence of about 15% [3] (Additional file 3). This large volume of HIV prevalence data was used to inform the fitting in Iran by ensuring that it generates a result that is in line with the trend described by the epidemiological data. In the absence of nationally-representative HCV prevalence data among PWID, we used the median, 25^th^ percentile, and 75^th^ percentile of all available HCV prevalence data in Iran, as identified in a recent systematic review of PWID in MENA [3], and therefore produced three predicted HIV epidemic time courses.

HIV prevalence at endemic equilibrium in Iran was also predicted directly, by subtracting the HCV threshold for sustainable HIV epidemic from each of the three HCV prevalence levels (25^th^, 50^th^, or 75^th^ percentile), then dividing by the deterministically model-estimated *RR*_*HCV*/*HIV*_ corresponding to each HCV prevalence level—that is using Eq. 3.

##### Effect of stochasticity

Since epidemic stochasticity could affect our modeling predictions by generating different HIV epidemic scales for the same HCV prevalence level, we examined the effect of stochasticity on our predictions of HIV prevalence at endemic equilibrium and also on *RR*_*HCV*/*HIV*_ and *OR*_*HCV*/*HIV*_ in Iran. We used the behavioral parameter values which correspond to the HCV prevalence level (25^th^, 50^th^, or 75^th^ percentile) that agrees most with the observed HIV prevalence data in Iran. We generated 5,000 stochastic epidemic simulations and calculated the mean value and 95% UI for the predicted HIV prevalence, *RR*_*HCV*/*HIV*_, and *OR*_*HCV*/*HIV*_ by fitting a log-normal distribution to the range of values.

##### Uncertainty analyses

We also examined the precision of our predictions of the HIV epidemic course in Iran. We conducted two separate multivariate biological and behavioral uncertainty analyses, using the methods described above, to specify the range of uncertainty in the predicted HIV prevalence, *RR*_*HCV*/*HIV*_, and *OR*_*HCV*/*HIV*_ that correspond to the HCV prevalence level that agrees most with the observed HIV prevalence data in Iran. At each run, the model was refit to this specific HCV prevalence.

## Results

### HCV to HIV infectiousness ratio

**Fig. 3:**
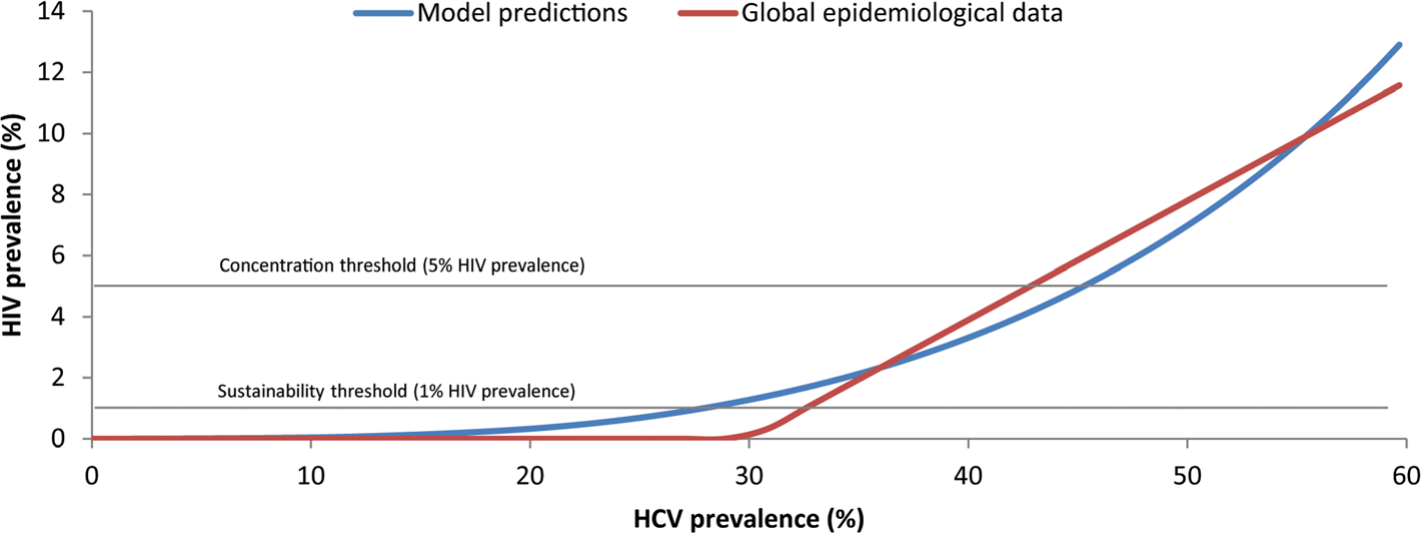
Relationship between HCV and HIV prevalence at endemic equilibrium among people who inject drugs, and model fitting to the segmented linear regression statistical model that summarizes the global epidemiological data as derived by Vickerman et al. [10]

The HCV and HIV model projections provided a good fit to the global epidemiological data describing the ecological association between HCV and HIV among PWID (Fig. 3). The optimum fitting value of the infectiousness ratio of HCV to HIV was found to be 7.8 (Additional file 2), suggesting that HCV is about eight times more transmissible per shared injection than HIV.

### Epidemiologic association and threshold effect

The fitted models were used to generate a broad spectrum of HCV and HIV prevalence and their epidemiological overlap (Fig. 3). As shown in Fig. 3, the predictions indicate a positive association between HCV and HIV prevalence at endemic equilibrium, with a threshold HCV prevalence of 27.9% for a sustainable HIV epidemic (>1%) and 45.2% for a concentrated HIV epidemic (>5%).

**Fig. 4:**
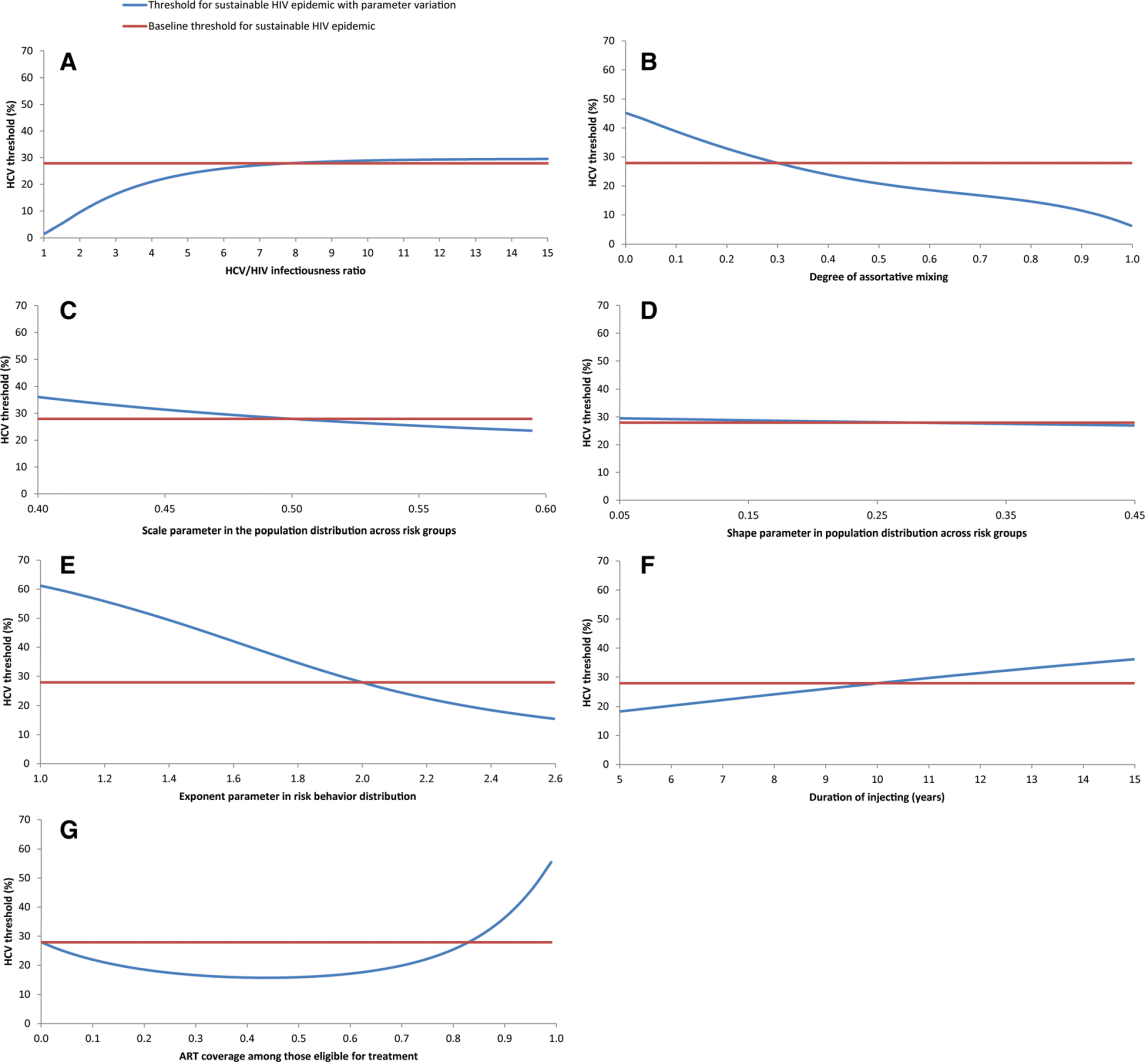
Sensitivity analyses on the HCV threshold for sustainable HIV epidemic (HIV prevalence >1%). These graphs illustrate the effect, on the HCV threshold for sustainable HIV epidemic, of the HCV/HIV infectiousness ratio (**a**), the degree of assortative mixing (**b**), the scale (**c**) and shape (**d**) parameters of the gamma distribution of the population across risk groups, the exponent parameter of the power law distribution of risk behavior (**e**), duration of injecting (**f**), and anti-retroviral therapy (ART) coverage (**g**). For each set of parameter values in these graphs, the average injecting risk behavior was varied; endemic HCV and HIV prevalence for each value of average injecting risk behavior were generated; and the HCV threshold for sustainable HIV epidemic was identified and plotted

**Fig. 5:**
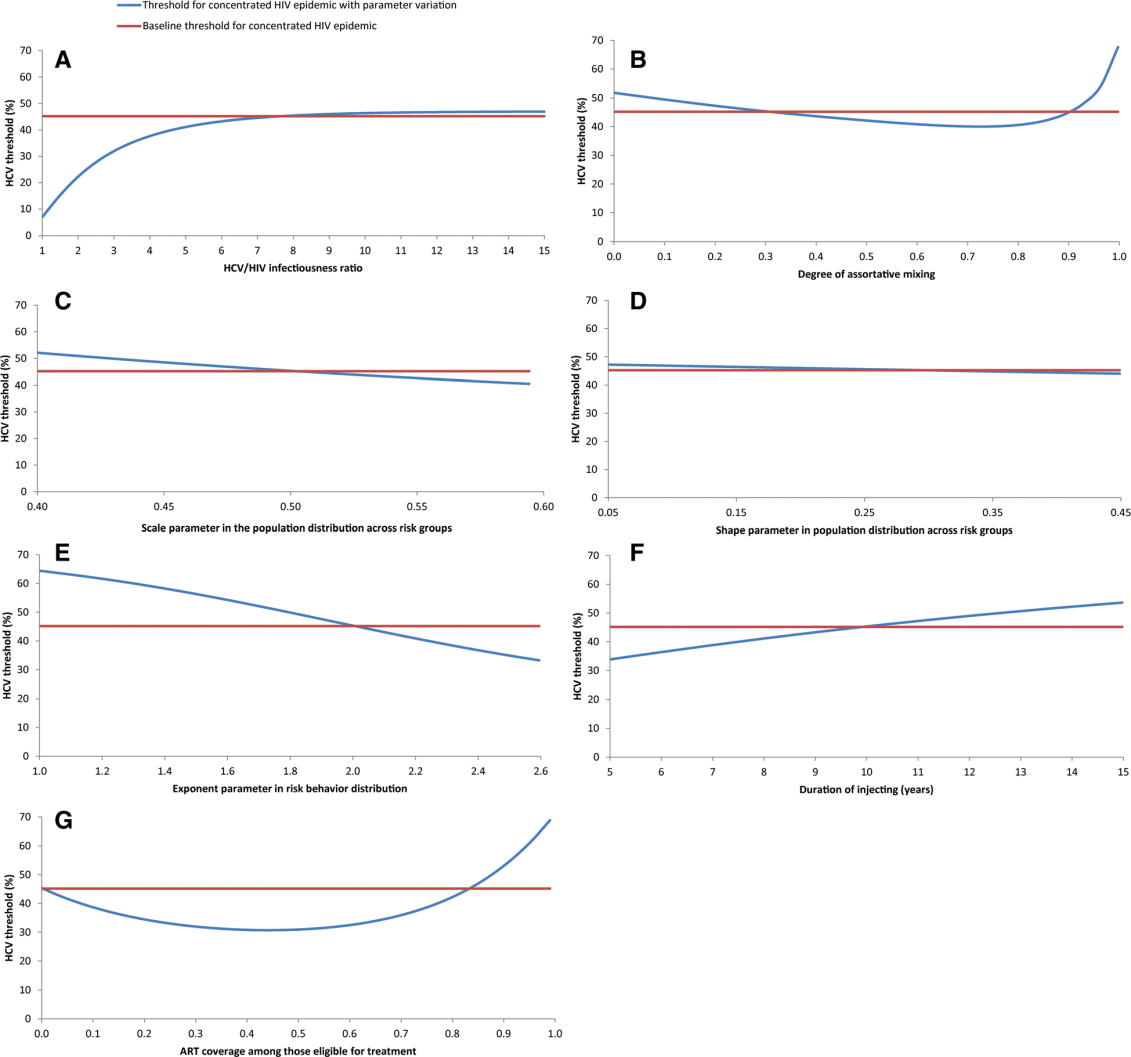
Sensitivity analyses on the HCV threshold for concentrated HIV epidemic (HIV prevalence >5%). These graphs illustrate the effect, on the HCV threshold for concentrated HIV epidemic, of the HCV/HIV infectiousness ratio (**a**), the degree of assortative mixing (**b**), the scale (**c**) and shape (**d**) parameters of the gamma distribution of the population across risk groups, the exponent parameter of the power law distribution of risk behavior (**e**), duration of injecting (**f**), and anti-retroviral therapy (ART) coverage (**g**). For each set of parameter values in these graphs, the average injecting risk behavior was varied; endemic HCV and HIV prevalence for each value of average injecting risk behavior were generated; and the HCV threshold for concentrated HIV epidemic was identified and plotted

Based on results of the sensitivity analyses (Figs. 4 and 5), changes in the HCV/HIV infectiousness ratio and in injecting risk behavior parameters within the specified wide ranges had a rather small effect on the existence or values of the HCV thresholds for sustainable and concentrated HIV epidemics. More specifically, there was a large reduction in the HCV thresholds only when the HCV/HIV infectiousness ratio was <7, while the effect of higher values of this ratio on the HCV thresholds was limited (Figs. 4 and 5 (a)). The HCV thresholds were mildly sensitive to changes in the degree of assortative mixing, except near the extremes of fully proportionate or fully assortative mixing (Figs. 4 and 5 (b)). Similarly, the HCV thresholds showed somewhat mild dependence to variation in the exponent parameter of the power law distribution of risk behavior, with a more pronounced effect when the exponent was closer to one (Figs. 4 and 5 (e)). There were small and limited effects, respectively, of the scale and shape parameters of the gamma distribution of the population across risk groups (Figs. 4 and 5 (c and d)). The duration of injecting and ART scale up had likewise a rather small effect on both thresholds. The effect of ART was most pronounced at very high coverage of above 90% (Figs. 4 and 5 (f and g)).

**Fig. 6:**
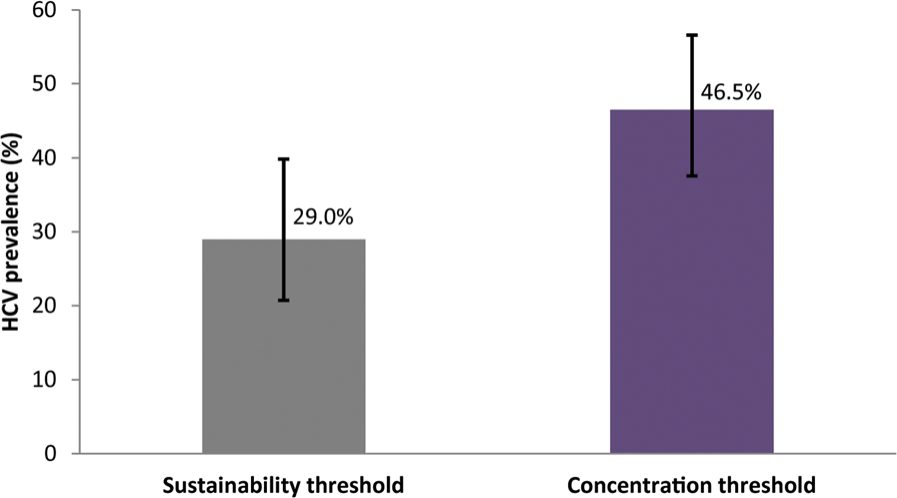
Estimated HCV thresholds for sustainable and concentrated HIV epidemic among PWID. The error bars represent the upper and lower bounds of the 95% uncertainty interval around the predicted HCV prevalence

Figure 6 shows our prediction and 95% UI of the HCV thresholds per the uncertainty analysis including all behavioral parameters. Results indicate a mean HCV threshold for sustainable and concentrated HIV epidemics of 29.0% (95% UI: 20.7-39.8) and 46.5% (95% UI: 37.6-56.6), respectively. The effect of the biological uncertainly on the HCV thresholds was smaller with a mean HCV prevalence of 27.5% (95% UI: 23.2-31.9) and of 44.7% (95% CI: 39.8-49.4) for sustainable and concentrated HIV epidemics, respectively.

### Overlapping epidemiology of HCV and HIV infections

**Fig. 7:**
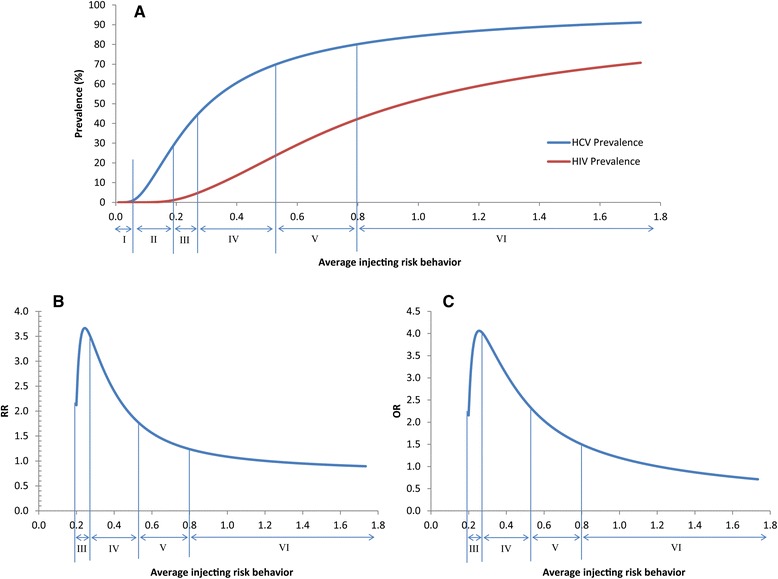
Epidemiological overlap between HCV and HIV infections among people who inject drugs. These graphs describe the epidemiological relationship between HCV and HIV infections by plotting endemic HCV and HIV prevalence (**a**), the risk ratio of endemic HCV to HIV prevalence (*RR*_*HCV/HIV*_) (**b**), and the odds ratio of endemic HCV to HIV prevalence (*OR*_*HCV/HIV*_) (**c**), as a function of the average injecting risk behavior (effective partnership change rate). Six epidemiological regimes linking HIV prevalence and HCV prevalence are discerned (**a**). The *RR*_*HCV/HIV*_ (**b**) and *OR*_*HCV/HIV*_ (**c**) are displayed for regimes III-VI with sustainable epidemics for both infections

The epidemiological overlap between HCV and HIV infections among PWID is illustrated in Fig. 7. Six dynamical epidemiological regimes were discerned based on the qualitative behavior of the prevalence of both infections, the *RR*_*HCV*/*HIV*_, and the *OR*_*HCV*/*HIV*_ (Fig. 7). The regimes are summarized in Table 1.

**Table 1:** Description of the dynamical regimes of the overlapping epidemiology of HCV and HIV infections among people who inject drugs

	HCV prevalence		HIV prevalence		*RR*_*HCV/HIV*_^ab^	*OR*_*HCV/HIV*_^ab^
	Range	Description	Range	Description	Range	Range
Regime I	<1%	Below sustainability	<1%	Below sustainability	..	..
Regime II	1–28%	Above sustainability - low scale HCV epidemic	<1%	Below sustainability	..	..
Regime III	28–45%	Large scale HCV epidemic	1–5%	Above sustainability threshold for HIV epidemic expansion and below concentration threshold	3.7-3.5	4.1-4.0
Regime IV	45–70%	Large scale HCV epidemic	5–24%	Concentrated HIV epidemic	3.5-1.8	4.0-2.3
Regime V	70–80%	Very large scale HCV epidemic	24–42%	Large scale HIV epidemic	1.8-1.2	2.3-1.5
Regime VI	>80%	Approaching maximum prevalence	>42%	Very large scale HIV epidemic and approaching maximum prevalence	<1.2	<1.5

^a^ We are reporting the range of *RR* and *OR* excluding those corresponding to the immediate vicinity of the HCV threshold for HIV sustainable transmission

^b^ The *RR*_*HCV/HIV*_ and *OR*_*HCV/HIV*_ are reported in descending order to reflect the decreasing trend in the ratios as a function of injecting risk behavior as per Fig. 7

In regime I, both HCV and HIV infections are below epidemic sustainability. In regime II, there is sustainable HCV transmission but HCV prevalence is low scale (below 30%); HIV is still below epidemic sustainability. In regime III, HCV prevalence is in the range of 28–45%. At this stage, HIV passed into epidemic sustainability but is below 5%, the threshold defining a concentrated HIV epidemic. In regime IV, HIV is in a concentrated state (prevalence 5–24%) since HCV prevalence is already above the threshold for a concentrated HIV epidemic (HCV prevalence >45%). In regime V, HCV prevalence is between 70 and 80%, and HIV prevalence is large scale, in the range of 25–42%. In regime VI where HCV prevalence is approaching maximum possible prevalence (100%), the HIV epidemic is very large scale and eventually also approaches maximum possible prevalence (100%) (Fig. 7, Table 1).

These six epidemiological regimes are also reflected in the trend and range of *RR*_*HCV*/*HIV*_ and *OR*_*HCV*/*HIV*_. These two measures of association become expressed only in regimes III-VI where both infections are above epidemic sustainability (Fig. 7, Table 1). In regime III, HCV epidemic expansion is substantially faster than that of HIV, resulting in *RR*_*HCV*/*HIV*_ and *OR*_*HCV*/*HIV*_ of 3.7-3.5 and 4.1-4.0, respectively. However, in subsequent regimes, HIV epidemic expansion gradually catches up with HCV epidemic expansion. This is reflected in the decreasing trend in *RR*_*HCV*/*HIV*_ and *OR*_*HCV*/*HIV*_ which reach 1.8 and 2.3 by the end of regime IV, respectively; and 1.2 and 1.5 by the end of regime V, respectively. In regime VI where both infections eventually reach maximum prevalence, the *RR*_*HCV*/*HIV*_ and *OR*_*HCV*/*HIV*_ ultimately reach their final asymptotic values (Fig. 7, Table 1).

### Predicting HIV epidemic scale using HCV prevalence: Effect of behavioral uncertainty

**Fig. 8:**
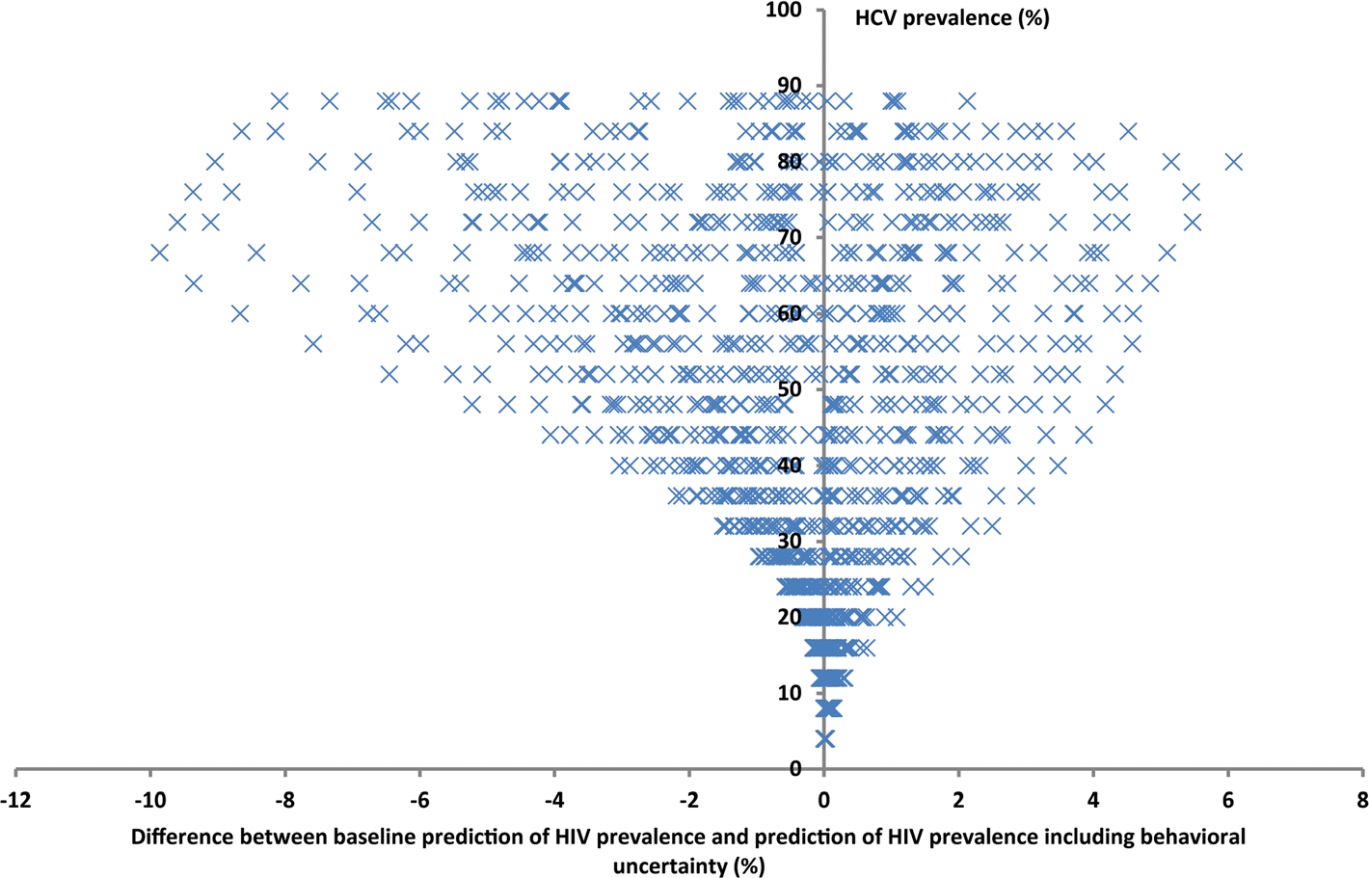
Effect of behavioral uncertainty on the HCV predictability of HIV epidemic expansion. The graph displays, for each HCV prevalence level, the difference between the baseline prediction of HIV prevalence and 50 random predictions of HIV prevalence, at this specific HCV prevalence level, that accommodate behavioral uncertainty

The effect of behavioral uncertainty on the prediction of HIV epidemic scale (HIV prevalence) across a wide range of HCV prevalence settings is shown in Fig. 8 and summarized by HCV/HIV dynamical regime in Table 2. Overall, 20% uncertainty in behavioral parameters resulted in a median of <1% and a maximum of <10% absolute HIV prevalence difference with the baseline prediction for HIV prevalence. The effect of behavioral uncertainty was negligible in regimes I & II and increased with higher regimes, until it reached a maximum in regimes IV and V with a median HIV prevalence difference of 1.9% (IQR: 0.9–3.4% and 0.9–3.8%, respectively) between the baseline prediction of HIV prevalence and the prediction including behavioral uncertainty (Fig. 8, Table 2).

**Table 2:** Effect of behavioral uncertainty on HCV predictions of HIV epidemic scale by HCV/HIV epidemiological regime

	HCV prevalence (%)	Predicted HIV prevalence (%)	HIV prevalence prediction difference (%)^a^	
	Range	Range	Median	IQR
Regime I	<1	<1	N/A	N/A
Regime II	1–28	<1	0.1	0.0-0.3
Regime III	28–45	1–5	1.1	0.6-1.7
Regime IV	45–70	5–24	1.9	0.9-3.4
Regime V	70–80	24–42	1.9	0.9-3.8
Regime VI	>80	>42	1.7	0.8-3.9
All regimes			0.9	0.2-2.2

*IQR* interquartile range

^a^ Difference between baseline prediction of HIV prevalence and prediction of HIV prevalence including behavioral uncertainty

### Case study: Iran

#### Predicting the time course of the HIV epidemic

**Fig. 9:**
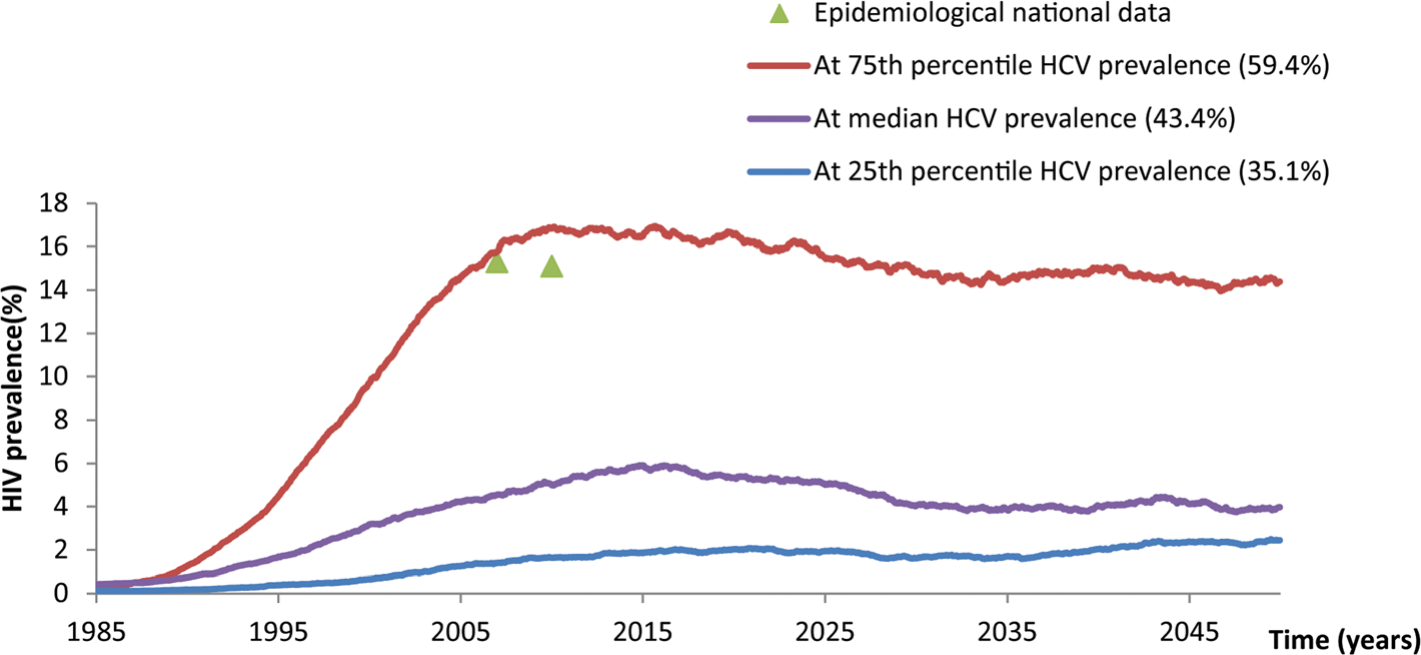
Representative stochastic simulations of HIV epidemic expansion at different HCV endemic prevalence levels among people who inject drugs in Iran, and comparison with epidemiological data

Based on available studies among PWID in Iran, the median HCV prevalence is 43.4% (interquartile range (IQR) 35.1–59.4%) [3]. The predicted time course of the HIV epidemic corresponding to each of these HCV prevalence levels is shown in Fig. 9 for three representative stochastic model runs. The predicted scale and time evolution of the HIV epidemic corresponding to the 75^th^ percentile of HCV prevalence agreed best with the actual time course of the HIV epidemic observed in Iran, whereby two rounds of nationally-representative surveillance surveys reported an HIV prevalence of 15.3% in 2006–7 [37] and 15.1% in 2010 [38]. An HIV prevalence at endemic equilibrium of 14.5% was predicted by the indicated stochastic run corresponding to 59.4% HCV prevalence (Fig. 9). The HIV stochastic predictions at the 25^th^ percentile and median HCV prevalence levels could not generate the observed HIV prevalence in Iran as they are below or just at the threshold for concentrated HIV epidemic, respectively.

To illustrate a simpler method for predicting HIV endemic prevalence using HCV prevalence, HIV prevalence at endemic equilibrium in Iran was also predicted using Eq. 3 involving the *RR*_*HCV*/*HIV*_ as derived from the deterministic modeling results presented above. As per Fig. 7, we predicted a *RR*_*HCV*/*HIV*_ of 2.5 for an observed HCV prevalence of 59% in Iran, resulting in predicted HIV prevalence of 13%. This value is comparable with the 15% HIV prevalence currently observed among PWID in this country [37, 38] and also with the value predicted above in a representative stochastic run (Fig. 9). Identical results were also obtained, as expected, when we used Eq. 4 in terms of the *OR*_*HCV*/*HIV*_ and its predicted value of 3.2 for HCV prevalence of 59% (Fig. 7).

#### Effect of stochasticity and behavioral and biological uncertainty on the predicted epidemic time course

The effect of stochasticity and of behavioral and biological uncertainty on our modeling predictions of endemic HIV prevalence, *RR*_*HCV*/*HIV*_, and *OR*_*HCV*/*HIV*_ in Iran corresponding to an HCV prevalence of 59.4% are shown in Additional file 4. Stochasticity generated a normal distribution of the natural log of HIV prevalence, *RR*_*HCV*/*HIV*_, and *OR*_*HCV*/*HIV*_, with a mean of 14.3% (95% UI: 13.0-15.5), 2.4 (95% UI: 2.2-2.6), and 3.1 (95% UI: 2.8-3.5), respectively. Uncertainty in behavioral parameters generated a skewed distribution of the natural log of HIV prevalence, *RR*_*HCV*/*HIV*_, and *OR*_*HCV*/*HIV*_, with a mean of 12.1% (95% UI: 5.4-21.1), 2.6 (95% UI: 1.5-5.8), and 3.3 (95% UI: 1.7-8.0), respectively. Uncertainty in biological parameters generated a normal distribution of the natural log of HIV prevalence, *RR*_*HCV*/*HIV*_, and *OR*_*HCV*/*HIV*_, with a mean of 13.5% (95% UI: 10.5–17.4%), 2.3 (95% UI: 1.8-3.0), and 2.9 (95% UI: 2.2-3.9), respectively.

## Discussion

Our mathematical modeling approach reproduced the epidemiologically-observed ecological association between HCV prevalence and HIV prevalence among PWID [10]. We estimated that HCV infection is 7.8 times more infectious per shared injection than HIV, and confirmed the existence of an HCV threshold for a sustainable HIV epidemic, which we estimated at 29.0%. We further estimated an HCV threshold of 46.5% for a concentrated HIV epidemic. These thresholds were independent of the uncertainty in biological parameters, and largely insensitive to the details of injecting risk behavior, except near extreme values of mixing pattern and variation in risk behavior among the different risk groups in a PWID population. The association between HCV and HIV was further described with six distinct dynamical regimes depicting the overlapping epidemiology of the two infections, and was quantified using defined and estimated measures of association.

Our main aim was to use this association between HCV and HIV to predict HIV epidemic potential using HCV prevalence. We showed, across a wide range of HCV prevalence settings, that behavioral uncertainty, arising from our limited knowledge of the details of the risk behavior environment in PWID, resulted in acceptable difference compared with our modeling predictions of HIV epidemic scale. This was demonstrated for data from Iran, where our modeling predictions reproduced the actual time course of the HIV epidemic, even in context of epidemic stochasticity and biological and behavioral uncertainty. All of these findings support our hypothesis that HCV prevalence can be used to make at least broad predictions of the future size of the HIV epidemic among PWID; and that these predictions can be further refined by applying mathematical models at specific HCV prevalence levels and potentially also for specific risk behavior environments.

HCV is known to be more infectious per percutaneous exposure than HIV, but the infectiousness ratio of the two infections via the parenteral route has not been estimated precisely. Evidence from needle-stick injury studies suggests that HCV is 4–10 times more transmissible per percutaneous injection than HIV [39–42]. By fitting our modeling approach to global epidemiological data for HCV and HIV prevalence, we provided, using a very different methodology, an independent estimate of the HCV to HIV infectiousness ratio among PWID at 7.8, which is in line with the above range.

The natural dynamics of our modeling approach generated a threshold behavior in the HCV-HIV association and estimated, in agreement with epidemiological data [10], a minimum HCV prevalence of about 30% below which HIV prevalence would be negligible. Earlier modeling work indicated that this threshold is dependent on the risk environment and thus likely to vary by setting, potentially explaining why some settings with similar HCV prevalence may have varying HIV prevalence levels [11, 13]. Because it is constrained by fitting to the epidemiologically-observed HCV-HIV association, our modeling did not lead to as wide variation in the value of the threshold. Despite accounting for uncertainty in biological and behavioral parameters, the HCV threshold was estimated within a reasonable range that is sufficient to inform policy and programming. Our modeling predicted that the HCV threshold of about 30% for a sustainable HIV epidemic is likely to apply in most global settings, except in unusual injecting settings where some risk behavior features are not typical of the common patterns of injecting networks.

One possible explanation for the diversity in HCV/HIV epidemics observed above the sustainability threshold could be that a number of the HIV epidemics globally may not have reached endemic equilibrium. Analysis of HCV-HIV epidemiological data among PWID has shown that the association between the two infections is strongest in settings of established HIV epidemics where HIV has reached an endemic level [9]. In MENA, for example, most HIV epidemics among PWID are emerging and have not reached endemic equilibrium [3]; this could be the case in other regions/settings where HIV prevalence levels are not typical of HCV-based predictions. Another possible explanation could be established harm reduction programs that are differentially effective in preventing HIV. This could be either due to the higher biological transmissibility of HCV compared to HIV, or because these programs were introduced after HCV reached endemic equilibrium but before HIV started its epidemic expansion. One such example is possibly Australia, a country with well-established needle-syringe exchange programs and where HIV prevalence has been low at about 1% despite HCV prevalence of 50–70% [43, 44].

Prioritization of affected populations for prevention interventions is tied to the potential for concentrated HIV epidemics (HIV prevalence >5%). We therefore derived the threshold HCV prevalence necessary for a concentrated HIV epidemic, which we estimated at about 45%. The effect of this threshold is manifested in settings of low intensity HIV epidemics where there is some HIV transmission among PWID, but the level of injecting risk behavior, as reflected by HCV prevalence of <45%, is not high enough to sustain concentrated HIV epidemics. The diverse HIV epidemic dynamics within Afghanistan provides an example of this threshold phenomenon (Table 3). HCV prevalence among PWID in Jalalabad and Mazar-i-Sharif (10–26%) is below the predicted sustainability threshold for HIV, and indeed HIV prevalence in these two cities has not exceeded 1% [45–47]. In Kabul however, where HCV prevalence at 28–37% is over the predicted sustainability threshold for HIV, but below the predicted concentration threshold for HIV, HIV prevalence, as expected, is in the range of 2–3% [45–47]. In Herat nonetheless, the high HCV prevalence of 49–70% is predicted to sustain a large concentrated HIV epidemic, and as expected, HIV prevalence has increased from 3% in 2007 to 13–18% in subsequent surveys [45, 46] (Table 3).

**Table 3:** Illustration of the threshold effects in Afghanistan

	Year	HCV prevalence (%)	Status	HIV prevalence (%)	Study
Jalalabad	2007	12.5	Below sustainability threshold	0.0	[47]
	2012	9.5		1.0	[46]
Mazar-i-Sharif	2007	24.1	Below sustainability threshold	0.0	[47]
	2009	25.5		1.0	[45]
	2012	18.8		0.3	[46]
Kabul	2005-6	36.6	Above sustainability threshold & below concentration threshold	3.0	[56]
	2008	36.1		2.1	[57]
	2009	37.1		3.2	[45]
	2012	27.6		2.4	[46]
Herat	2007	49.1	Above concentration threshold	3.2	[47]
	2009	57.9		18.2	[45]
	2012	70.0		13.3	[46]

Six epidemiological regimes incorporating the HCV thresholds were discerned in describing the overlapping epidemiology of HCV and HIV infections among PWID (Figs. 7 and 8). In the first regime, the level of injecting risk behavior is extremely low and does not sustain an HCV nor HIV epidemic. In the second regime, the level of injecting risk behavior is relatively low, but enough to sustain a low scale HCV epidemic. With HIV being eight times less transmissible than HCV, an injecting risk network in this regime cannot sustain an HIV epidemic; HIV spreads slowly and inefficiently at very low level and is sensitive to stochastic fluctuations. In the third regime, the level of risk behavior is above the threshold needed to maintain a sustainable HIV epidemic, but is still not high enough to maintain an HIV prevalence larger than 5%. In the fourth and apparently most common regime globally [10], the level of risk behavior is substantial, as reflected by an HCV prevalence of 45–70%, and this level of risk behavior is large enough to maintain concentrated HIV epidemics reaching up to about 25% in HIV prevalence. In the fifth regime, HCV prevalence increases very slowly with risk behavior as it has already attained extreme values reflecting infection transmission saturation (>70%). However, HIV prevalence increases substantially even with very small increments in HCV prevalence, resulting in large scale HIV epidemics. In the last regime, HCV continues its very slow growth with risk behavior, while HIV is still growing noticeably, though at slower pace compared with the fourth regime. In this last regime, both infections eventually reach maximum possible prevalence.

In sum, HIV epidemic behavior in a PWID population can be broadly predicted based on the regime HCV prevalence belongs to. Any public health intervention aiming at reducing injecting risk behavior among PWID (such as education and awareness programs) may, if successful, shift endemic HCV and HIV prevalence levels to a new regime where HCV will still be predictive of HIV but according to the HCV-HIV association characterized by the new regime (Fig. 7).

The boundaries of each of these six epidemiological regimes were further described in our study by the *RR*_*HCV*/*HIV*_ and *OR*_*HCV*/*HIV*_, which were defined and estimated to quantify the association between HCV and HIV infections. The ranges of *RR*_*HCV*/*HIV*_ and *OR*_*HCV*/*HIV*_ in each regime were found to be relatively narrow. For example, in the fourth regime where a large fraction of the HIV PWID epidemics worldwide belongs [10], the *RR*_*HCV*/*HIV*_ and *OR*_*HCV*/*HIV*_ are relatively stable and hover around a value of 3 (Fig. 7b and c, Table 1). This indicates that when HCV prevalence is in the range of 45–70%, HIV prevalence will be about three times smaller than the observed HCV prevalence minus 27.9%, the threshold HCV prevalence for a sustainable HIV epidemic (Eq. 3). We found that behavioral uncertainty, and to a lesser extent biological uncertainty and stochasticity, could affect the predicted values of the *RR*_*HCV*/*HIV*_ and *OR*_*HCV*/*HIV*_, but overall in a predictable way that does not undermine their potential programmatic use to characterize HCV-HIV overlapping epidemiology and to make predictions of future HIV epidemic scale.

Our findings provide a rationale for using HCV prevalence as a predictor of future HIV epidemic scale in PWID. This approach provides also several specific prediction methods with different levels of precision. First, the derived HCV thresholds can be used in conjunction with observed HCV prevalence to predict, in a broad term, whether a PWID population is likely or not to experience a sustainable or concentrated HIV epidemic (such as in the example of Table 3). Second, linking a PWID population with its epidemiological regime based on HCV prevalence, that is one of the six epidemiological regimes in Table 1, can provide a range for the predicted future HIV prevalence. For example, a PWID population with an HCV prevalence of 50% belongs to regime IV and is therefore likely to experience an HIV epidemic with an HIV prevalence in the range of 5–24%. Third, a more precise HIV prevalence range can be estimated using the derived *RR*_*HCV*/*HIV*_ (or *OR*_*HCV*/*HIV*_) range for each specific epidemiological regime and Eq. 3 (or Eq. 4). For example, the *RR*_*HCV*/*HIV*_ in regime IV ranges between 1.8 and 3.5. For a PWID population at 50% HCV prevalence, this *RR*_*HCV*/*HIV*_ range translates, through Eq. 3, into a predicted HIV prevalence range of 6–12%. Finally, the most precise estimation of HIV epidemic scale can be obtained by applying the mathematical models directly at a specific HCV prevalence. For a 50% HCV prevalence, modeling predicts an HIV prevalence of 7%. Uncertainty analyses can be also conducted on the later estimate, using also the models, to provide an uncertainty interval for this estimate, as was done for Iran above.

There were several limitations in our study. Although we used an elaborate mathematical model structure, we may not have captured some of the complexities of injecting risk networks and of HCV/HIV dynamics. For example, we did not allow movement of PWID between different risk groups, and did not incorporate the effect of HCV-HIV co-infection and its potential effect on HCV transmission and spontaneous clearance. We also did not consider the sexual transmission of HIV since its relative importance among PWID is small except among specific sub-populations of PWID who are also men who have sex with men or female sex workers [3, 10, 39, 48]. In most of MENA settings where our applications are aimed at, the HIV epidemics are characterized by limited sexual HIV transmission not only among PWID, but even among populations at high risk of HIV sexual transmission such as men who have sex with men and female sex workers and their clients [49–52]. It is therefore unlikely that sexual transmission of HIV would affect our results and their applications in MENA. Our model could be extended in future research to include sexual HIV transmission where it would be of value to estimate the relative contribution of sexual versus parenteral HIV transmission. This would be especially relevant for applications of the methodology to settings other than MENA where sexual transmission may be more prominent.

The HIV model did not include scale-up of ART, but ART coverage was included in a sensitivity analysis where it had overall a minor effect on the HCV thresholds except near extreme values of coverage (>90%). Currently MENA has the lowest ART coverage of all regions globally at 17% [30], with unpublished data suggesting even lower coverage among PWID (World Health Organization, unpublished). Similarly, and with the very low coverage among PWID [53], the model did not include the effect of HCV treatment. However, with the newly available direct-acting antivirals to treat HCV, scale-up of HCV treatment among PWID is expected to increase, thus possibly influencing HCV transmission dynamics and complicating the relationship between HIV and HCV. In this case, our models would need to be extended to capture HCV treatment scale up and coverage, in addition to uneven healthcare services among PWID, to adjust the modeling predictions of future HIV prevalence based on existing HCV prevalence levels.

Our modeling predictions can be also constrained by limitations in the data input of our models. Nevertheless, a wide range was attached to the biological and behavioural parameters to capture the uncertainty in our knowledge of these parameters. We also fitted our modeling predictions to available global epidemiological data [10] to derive key parameters that are not precisely measured, mainly the HCV/HIV infectiousness ratio. While admittedly the epidemics in different global settings could be in different stages, and not all epidemics of substantial HCV and HIV prevalence are at equilibrium, we fitted to all global data rather than to the temporal trend of the epidemic in a specific setting, since the main purpose was to derive this biological and not setting-specific parameter. Also, fitting our dynamical model to the actual epidemiological data and their time series would have been superior to fitting to the regression line that summarizes these data [10]. Finally, predictions of HIV epidemic scale using the methodology we propose will depend on the quality and representativeness of HCV prevalence data, in the same way any other prediction method is dependent on the quality of input data. This highlights the importance of collecting quality and representative HCV prevalence data among PWID.

Despite these limitations, we structured our models through a parsimonious approach to ensure that the models complexity is constrained by the available data, and predictions are robust even with broad ranges in parameter values. Biological parameters in our model are generally obtained from primary and most recent empirical data, similar to comparable mathematical modeling studies in the literature. Admittedly, behavioral parameters have more uncertainty but the extensive sensitivity and uncertainty analyses we conducted indicated overall a minor effect on our model’s predictions of HCV thresholds and endemic HIV prevalence. Our model further fitted well the global epidemiological data and was able to reproduce a number of the findings of previous ecological and modeling work [9–11, 13].

Our findings, and the concept we present, have important policy, programming, and resource allocation implications. We demonstrated that HCV can be used to predict future HIV epidemics and their scale. Because of stochasticity and biological and behavioural uncertainty, predictions may not be very precise in terms of the exact HIV prevalence foreseen. However even with coarse predictions, such an approach can be effective in pinpointing settings that are likely to experience substantial HIV epidemics in the future, and accordingly need to be prioritized for prevention interventions. In this sense, HCV acts as a *temperature scale* of the level of risk behavior in an injecting network, and can be used as an index to measure the risk and severity of potential HIV epidemics among PWID. This is, in essence, a population-based diagnostic test or a screening approach, similar to other individual-based diagnostic tests or public health screening programs, such as for heart disease or breast cancer. While such diagnostics or screening programs may not have perfect sensitivity or specificity for the disease of interest, they have an important public health impact by averting or controlling disease through early detection. Moreover, studies have shown that identifying and targeting most-at-risk populations significantly improve the cost-effectiveness of interventions [1]. For example, a recent study has indicated that by dividing the population into two groups of high and low risk behavior, targeting those at higher risk of acquiring HIV would increase the effectiveness of an intervention (voluntary medical male circumcision) ten-fold [54, 55]. By dividing the population into six risk groups, the intervention becomes 80 times more effective if the highest risk group is targeted compared to targeting the lowest risk group [54, 55].

## Conclusions

We investigated and characterized the poorly-understood association between HCV and HIV infections among PWID. The overlapping epidemiology of the two infections was described using distinct dynamical regimes and quantified with devised measures of association. Despite the complexity of the models and of the HCV-HIV association, these measures offered a simple applied tool for policy makers and program officers to predict HIV epidemic potential in PWID populations. We also proposed several methods with varying levels of precision for predicting HIV epidemic scale, and this concept was demonstrated in a specific country, Iran. The methodology proposed in the present study has a practical relevance which can be disseminated directly at the level of national stakeholders or in consultation with the international organizations leading the HIV/HCV response in the region, namely the World Health Organization, Joint United Nations Programme on HIV/AIDS, and the World Bank.

By identifying and targeting settings where HIV prevalence among PWID is currently at low level, but where the level of risk behavior as reflected by HCV prevalence is indicative of substantial future HIV epidemics, the proposed methodology not only helps in prioritization of PWID populations with high HIV epidemic potential, but also will lead to higher cost-effectiveness of HCV/HIV interventions. This is particularly critical in resource-limited settings, such as MENA and other regions in the world.

## Additional files

Additional file 1: Mathematical models description. (DOCX 302 kb)
Additional file 2: Models assumptions in terms of parameter values. (DOCX 95 kb)
Additional file 3: Trend of HIV prevalence among PWID in Iran as described by available HIV point-prevalence measures 1990–2013. (TIF 1115 kb)
Additional file 4: Effect of stochasticity (purple) and of behavioral (blue) and biological (red) uncertainty on the modeling predictions of the endemic HIV prevalence, *RR*_*HCV/HIV*_ and *OR*_*HCV/HIV*_ at 59.4% HCV prevalence in Iran. (TIF 3208 kb)

## Abbreviations

ART: Antiretroviral therapy
HCV: Hepatitis C virus
IQR: Interquartile range
MENA: Middle East and North Africa
*OR*_*HCV/HIV*_: Odds ratio of HCV prevalence to HIV prevalence
PWID: People who inject drugs
*RR*_*HCV/HIV*_: Risk ratio of HCV prevalence to HIV prevalence
UI: Uncertainty interval
