# Supplementary material for: HCV prevalence can predict HIV epidemic potential among people who inject drugs: mathematical modeling analysis
Source: BMC Public Health. 2016 Dec 3;16:1216. doi: 10.1186/s12889-016-3887-y (PMC5135754; doi:10.1186/s12889-016-3887-y)
Supplement: Additional file 1: — Mathematical models description. (DOCX 302 kb) [file 12889_2016_3887_MOESM1_ESM.docx]

**Text S1**

**Mathematical models description**

1. **HIV model structure**

We developed a deterministic/stochastic compartmental mathematical model that describes the parenteral transmission of HIV through sharing unsterile needles/syringes among people who inject drugs (PWID) (Figure 1). Other methods of HIV transmission (e.g. sexual) are not considered in the model. The model stratifies the PWID population into compartments according to HIV status, stage of HIV infection, and level of injecting risk behavior.

Susceptible

*µ+ƞ*

*µ*

*µ+ƞ*

$$I_{i,1}$$

Acute

infection

Latent infection

*µ+ƞ*

Advanced infection

*µ+ƞ*

**Figure S1.** HIV model structure

The model was solved both deterministically and stochastically. The deterministic version of the model was expressed with the below system of coupled nonlinear differential equations for each risk group. For the stochastic version, we used the same transition rates in this deterministic system of equations to generate the stochastic process.

$\frac{dS_{i}}{dt}=\mu N_{0,i}-(\mu+\eta)S_{i}-\Lambda_{i}S_{i}$

$\frac{dI_{i,1}}{dt}=\Lambda_{i}S_{i}-(\mu+\eta)I_{i,1}-\omega_{1}I_{i,1}$

$$\frac{dI_{i,2}}{dt}=\omega_{1}I_{i,1}-(\mu+\eta)I_{i,2}-\omega_{2}I_{i,2}$$

$$\frac{dI_{i,3}}{dt}=\omega_{2}I_{i,2}-(\mu+\eta)I_{i,3}-\omega_{3}I_{i,3}$$

To accommodate heterogeneity of injecting risk behavior, we stratified the population into 7 injecting risk groups, defined with the index $i$ ($i=1,2,...,7$ representing the low to high risk groups). Here $S_{i}$ is the HIV susceptible population in the $i$–risk group, and $I_{i,\beta}$ is the HIV infected population in the $i$–risk group. The index $\beta$ marks the stage of HIV pathogenesis; $\beta=1,2,3$ represent the acute, latent, and advanced stages, respectively.$N_{0,i}$is the initial population size of each $i$-risk group.$\mu$ is the natural mortality rate, and $\eta$ is the leaving injecting career rate. The rate of progression from one HIV stage to the next is described by $\omega_{1}$ and$\omega_{2}$, while $\omega_{3}$ is the rate of HIV/AIDS disease mortality. The rate $\Lambda_{i}$ is the HIV force of infection (incidence rate of infection) experienced by the $S_{i}$ susceptible population.$\Lambda_{i}$ is given by:

$$\Lambda_{i}=\rho_{S_{i}}\sum_{j=1}^{7} \sum_{\beta=1}^{3} t_{I_{j,\beta}\to S_{i}}G_{i,j}\frac{\rho_{I_{j,\beta^{'}}}I_{j,\beta^{'}}}{\rho_{S_{j}}S_{j}+\sum_{\beta^{'}=1}^{3} \rho_{I_{j,\beta^{'}}}I_{j,\beta^{'}}}$$

where $\rho_{P_{i}}$describes the *effective* new partner acquisition rate for any population variable $P_{i}$ ($S_{i}$or $I_{i}$) (note further discussion in section 3.2 below).

The parameter $t_{I_{j,\beta}\to S_{i}}$ defines the HIV transmission probability per partnership between a member of the susceptible population $S_{i}$ and a member of the HIV infected population $I_{j,\beta}$:

$$t_{I_{j,\beta}\to S_{i}}=1-\left(1-p_{I_{j,\beta}\to S_{i}}^{HIV}\right)^{n_{I_{j,\beta}\leftrightarrow S_{i}}}$$

It is expressed in terms of HIV transmission probability per needle/syringe sharing act per HIV stage in this partnership ($p_{I_{j,\beta}\to S_{i}}^{HIV}$) and the number of needle/syringe sharing acts per partnership ($n_{I_{j,\beta}\leftrightarrow S_{i}}$).

The mixing among the different risk groups is dictated by the injecting-mixing matrix $G_{i,j}$. This matrix provides the probability that an individual in risk group $i$ would choose a partner in risk group $j$. It is given by:

$$G_{i,j}=e\theta_{i,j}+\left(1-e\right)\frac{\rho_{S_{j}}S_{j}+\sum_{\beta=1}^{3} \rho_{I_{j,\beta}}I_{j,\beta}}{\sum_{k=1}^{7} \left(\rho_{S_{k}}S_{k}+\sum_{\beta^{'}=1}^{3} \rho_{I_{k,\beta^{'}}}I_{k,\beta^{'}}\right)}$$

Here, $\theta_{i,j}$ is the identity matrix and the parameter $e\in\left[0,1\right]$ measures the degree of assortativeness in the mixing. At the extreme $e=0$, the mixing is proportionate (choosing partners with no preferential bias based on the kind of risk group) while at the other extreme $e=1$, the mixing is fully assortative as individuals choose partners only from within their own risk group [[1](#_ENREF_1)].

1. **HCV Model structure**

We developed a deterministic/stochastic compartmental mathematical model that describes the parenteral transmission of HCV through sharing unsterile needles/syringes among PWID (Figure 2). The model is similar in structure to the HIV model described above, and also stratifies the PWID population into compartments according to HCV status and stage of infection, and level of injecting risk behavior.

Susceptible

*µ+ƞ*

*µ*

*µ+ƞ*

*δ_1_*

$$Y_{i,1}$$

Acute

infection

*µ+ƞ*

*γ_1_*

*1-γ_1_*

*µ+ ƞ*

$$Y_{i,2}$$

Chronic

infection

$$X_{i}^{2}$$

Susceptible

(Previously exposed)

*µ+ƞ*

*δ_2_*

*γ_2_*

*1-γ_2_*

Secondary acute infection

**Figure S2.** HCV model structure

The model was solved both deterministically and stochastically. The deterministic version of the model was expressed in terms of a system of coupled nonlinear differential equations for each risk group:

$$\frac{dX_{i}^{1}}{dt}=\mu N_{0,i}-(\mu+\eta)X_{i}^{1}-\lambda_{X_{i}^{1}}X_{i}^{1}$$

$$\frac{dY_{i,1}}{dt}=\lambda_{X_{i}^{1}}X_{i}^{1}-\left(\mu+\eta\right)Y_{i,1}-\delta_{1}Y_{i,1}$$

$$\frac{dY_{i,2}}{dt}=\delta_{1}\left(1-\gamma_{1}\right)Y_{i,1}+\delta_{2}\left(1-\gamma_{2}\right)Y_{i,3}-\left(\mu+\eta\right)Y_{i,2}$$

$$\frac{dX_{i}^{2}}{dt}=\delta_{1}\gamma_{1}Y_{i,1}+\delta_{2}\gamma_{2}Y_{i,3}-\left(\mu+\eta\right)X_{i}^{2}-\lambda_{X_{i}^{2}}X_{i}^{2}$$

$$\frac{dY_{i,3}}{dt}=\lambda_{X_{i}^{2}}X_{i}^{2}-\left(\mu+\eta\right)Y_{i,3}-\delta_{2}Y_{i,3}$$

The index $i$ defines the seven injecting risk groups, ($i=1,2,...,7$ representing the low to high risk groups). Here $X_{i}^{1}$ is the HCV susceptible population in the $i$–risk group, and $Y_{i,\beta}$ is the HCV infected population in the $i$–risk group. The index $\beta$ marks the stage of HCV pathogenesis; $\beta=1,2,3$ represent the acute, chronic, and secondary acute stages, respectively. $X_{i}^{2}$ is the population, in the $i$–risk group, that was previously exposed to HCV infection but cleared it and is now susceptible for HCV reinfection. $N_{0,i}$ is the initial population size of each $i$-risk group. $\mu$ is the natural mortality rate, and *ƞ* is the leaving injecting career rate. The rates of progression from primary and secondary acute HCV infections are $\delta_{1}$ and$\delta_{2}$, respectively. $\gamma_{1}$ is the percentage of primary HCV infections that clear, and $\gamma_{2}$ the percentage of HCV reinfections that clear.

The rate $\lambda_{X_{i}^{1}}$ is the HCV force of infection experienced by the $X_{i}^{1}$ susceptible population and is given by:

$$\lambda_{X_{i}^{1}}=\rho_{X_{i}^{1}}\sum_{j=1}^{7} \sum_{\beta=1}^{3} R_{I_{j,\beta}\to X_{i}^{1}}H_{i,j}\frac{\rho_{Y_{j,\beta^{'}}}Y_{j,\beta^{'}}}{\rho_{X_{i}^{1}}X_{i}^{1}+\rho_{X_{i}^{2}}X_{i}^{2}+\sum_{\beta^{'}=1}^{3} \rho_{Y_{j,\beta^{'}}}Y_{j,\beta^{'}}}$$

We assume here that the susceptible population $X_{i}^{2}$ experiences the same force of infection as the $X_{i}^{1}$ population (no acquired immunity):

$$\lambda_{X_{i}^{2}}=\lambda_{X_{i}^{1}}$$

$\rho_{P_{i}}$ describes the *effective* new partner acquisition rate for any population variable $P_{i}$ ($X_{i}$or $Y_{i}$) (note further discussion in section 3.2 below).

The parameter $R_{Y_{j,\beta}\to X_{i}^{1}}$ defines the HCV transmission probability per partnership between a member of the susceptible population $X_{i}^{1}$ and a member of the HCV infected population $Y_{i,\beta}$:

$$R_{Y_{j,\beta}\to X_{i}^{1}}=1-\left(1-p_{Y_{j,\beta}\to X_{i}^{1}}^{HCV}\right)^{n_{Y_{j,\beta}\leftrightarrow X_{i}^{1}}}$$

It is expressed in terms of HCV transmission probability per needle/syringe sharing act per HCV stage in this partnership $p_{Y_{j,\beta}\to X_{i}^{1}}^{HCV}$ and the number of needle/syringe sharing acts per partnership ($n_{Y_{j,\beta}\leftrightarrow X_{i}^{1}}$),

The mixing among the different risk groups is dictated by the mixing matrix $H_{i,j}$. This matrix provides the probability that an individual in risk group $i$ would choose a partner in risk group $j$. It is given by:

$$H_{i,j}=e\theta_{i,j}+\left(1-e\right)\frac{\rho_{X_{j}^{1}}X_{j}^{1}+\rho_{X_{j}^{2}}X_{j}^{2}+\sum_{\beta=1}^{3} \rho_{Y_{j,\beta}}Y_{j,\beta}}{\sum_{k=1}^{10} \left(\rho_{X_{j}^{1}}X_{j}^{1}+\rho_{X_{j}^{2}}X_{j}^{2}+\sum_{\beta^{'}=1}^{3} \rho_{Y_{j,\beta^{'}}}Y_{j,\beta^{'}}\right)}$$

Here, $\theta_{i,j}$ is the identity matrix and the parameter $e\in\left[0,1\right]$ measures the degree of assortativeness in the mixing. At the extreme$e=0$, the mixing is proportionate while at the other extreme $e=1$, the mixing is fully assortative [[1](#_ENREF_1)].

1. **Injecting risk behavior**
   1. *Distribution of injecting risk behavior in the population*

The PWID population was stratified into a number of risk groups. In the absence of direct empirical data to inform on the exact distribution of injecting risk behavior in a given PWID population, we assumed that the proportion of the PWID population initially in each risk group $i$ follows a gamma distribution. This assumption was informed by previous theoretical work [[2-5](#_ENREF_2)] and mathematical modeling of HIV sexual transmission [[6](#_ENREF_6), [7](#_ENREF_7)], and accommodates wider flexibility [[4](#_ENREF_4)]. The gamma distribution of the population size across the risk groups is given by:

$$p(i)=\frac{1}{b^{a}\Gamma(a)}i^{a-1}e^{\frac{-i}{b}}$$

Here $a$ is the shape parameter determined through normalization of the distribution, and $b$ is the scale parameter in the gamma distribution.

- 1. *The effective new injecting partner acquisition rate*

The $\rho_{P_{i}}$ parameter describes the number of new injecting partners an individual in a specified risk group acquires, but also effectively other factors that enhance the risk of exposure to the infection such as concurrency and clustering within injecting networks, and variability in injecting risk behavior in the population. Since the exact nature of injecting behavior and injecting networks is not well-understood and varies within and across communities, $\rho_{P_{i}}$ is effectively a summary measure of the population-specific level of injecting risk behavior, and captures the distribution and strength of the risk of exposure to HIV (or HCV) infection. The form of the $\rho_{P_{i}}$ distribution across different risk groups was defined through a power law function as:

$\rho_{P_{i}}\text{ }=Ci^{\alpha}$ where $\alpha$ is the exponent in the power-law function and $C$ is an overall constant.

This form is motivated by simulations using an individual-based network model developed to explore the diversity of risk in risk networks [[8](#_ENREF_8)], and also by analyses of the architecture of complex weighted networks [[9](#_ENREF_9), [10](#_ENREF_10)], and by an analysis of the average separation between individuals in a network or a sub-network [[11](#_ENREF_11), [12](#_ENREF_12)]. The latter can be seen as a proxy of the size of the “ecology” through which an individual can acquire an infection. Here $C$is a constant determined by the average risk behavior and $\alpha$ is the exponent parameter that determines the level of variability in the effective new injecting partner acquisition rate [[8](#_ENREF_8)].

1. **Parameter values**

The parameters of the model were derived using current empirical data on HIV/HCV epidemiology and natural history, and are listed in Table S1 along with their references.

We assumed that the transmission probability of HIV per sharing needle/syringe is 10 times higher than the probability of transmission per coital act in each HIV stage [[13](#_ENREF_13)]. The latter were based on recent re-analyses of the Rakai Study data [[14-17](#_ENREF_14)]. The durations of the acute, latent, and advanced stages of HIV infection were assumed to be 49 days, 9 years, and 2 years, respectively. These choices were based on compilation of data by UNAIDS indicating that the average duration from HIV acquisition to death, in absence of antiretroviral therapy, is about 11 years [[18](#_ENREF_18)], and based on the classification in Wawer *et al.* [[17](#_ENREF_17)], re-analysis of the Rakai data for acute infection [[14](#_ENREF_14)], and measured time from seroconversion to death in several cohort studies [[19](#_ENREF_19), [20](#_ENREF_20)].

The transmission probability of HCV in the chronic stage was calculated based on the transmission probability of HIV in the latent stage [[14-17](#_ENREF_14)]. Based on model fitting to empirical data (please see main text), we found HCV transmission probability in the chronic stage to be 7.8 times greater than that of HIV. HCV transmission probability in the primary acute infection was estimated earlier at 2.7 times HCV transmission probability in the chronic stage [[21](#_ENREF_21)]. We also assumed that HCV transmission probability in the secondary acute stage is half of that in the primary acute stage, based on the 50% reduction in viral load following reinfection compared to primary infection, as estimated in a cohort study by Obsurn et al. [[22](#_ENREF_22)]. The durations of primary and secondary HCV acute stages were assumed to be 16.5 weeks and 4.1 weeks, respectively, based on direct measurement in recent prospective cohort studies [[22](#_ENREF_22), [23](#_ENREF_23)]. The percentage of primary HCV infections and HCV reinfections that clear were assumed to be 25% and 83%, respectively, based on cohort studies data [[22](#_ENREF_22), [23](#_ENREF_23)].

As for the parameters of injecting risk behavior, the degree of assortativeness ($e$) was fixed at 0.3; a representative value informed by earlier modeling work on HIV [[16](#_ENREF_16)]. Meanwhile, the scale ($b$), and shape ($a$) parameters (in the gamma distribution of the population across the different risk groups) were fixed at 0.5 and 0.28, respectively, based on our model fitting to a statistical model summarizing the HCV/HIV empirical data [[24](#_ENREF_24)]. The exponent parameter in the power law function of the distribution of injecting risk behavior ($\alpha$) was fixed at 2.0, based also on our model fitting to HCV/HIV empirical data [[24](#_ENREF_24)]. The duration of the injecting career was assumed to be 10 years as informed by empirical data [[13](#_ENREF_13)].

1. **Effect of ART - sensitivity analysis**

We assessed the sensitivity of the HCV thresholds for HIV epidemic expansion to antiretroviral therapy (ART) scale up. We assumed that the efficacy of ART in reducing HIV transmission among PWID is 100%, based on clinical trials of treatment for prevention and other observational data [[25](#_ENREF_25), [26](#_ENREF_26)]. Accordingly, the probability of HIV transmission per needle/syringe sharing act ($p_{I_{j,\beta}\to S_{i}}^{HIV}$) in a population with ART coverage of among those eligible for treatment is reduced by a factor of . We assumed that with ART scale up, all infected PWID in the advanced stage and half of those in the latent stage would be eligible for ART treatment, which corresponds roughly to an eligibility treatment criteria of CD4 cell count < 500 cells/μl [[27](#_ENREF_27)].

We also assumed that ART slows disease progression from onset of infection to death. The average duration of latent infection in the HIV infected population up to treatment initiation was assumed to be . The average duration from treatment initiation to death was assumed to be. Accordingly, 100% coverage among those eligible will double the average duration from onset of infection to death in the HIV infected population from 11 years to 22 years.

**REFERENCES**

1. Garnett GP, Anderson RM. Factors controlling the spread of HIV in heterosexual communities in developing countries: patterns of mixing between different age and sexual activity classes. Philos Trans R Soc Lond B Biol Sci. 1993;342(1300):137-59. Epub 1993/10/29. doi: 10.1098/rstb.1993.0143. PubMed PMID: 7904355.

2. Anderson RM, Medley GF, May RM, Johnson AM. A preliminary study of the transmission dynamics of the human immunodeficiency virus (HIV), the causative agent of AIDS. IMA journal of mathematics applied in medicine and biology. 1986;3(4):229-63. Epub 1986/01/01. PubMed PMID: 3453839.

3. Hamilton DT, Handcock MS, Morris M. Degree distributions in sexual networks: a framework for evaluating evidence. Sexually transmitted diseases. 2008;35(1):30-40. PubMed PMID: 18217224.

4. Handcock MS, Jones JH. Likelihood-based inference for stochastic models of sexual network formation. Theoretical population biology. 2004;65(4):413-22. doi: 10.1016/j.tpb.2003.09.006. PubMed PMID: 15136015.

5. Ghani AC, Garnett GP. Risks of acquiring and transmitting sexually transmitted diseases in sexual partner networks. Sex Transm Dis. 2000;27(10):579-87. Epub 2000/12/01. PubMed PMID: 11099073.

6. Awad SF, Abu-Raddad LJ. Could there have been substantial declines in sexual risk behavior across sub-Saharan Africa in the mid-1990s? Epidemics. 2014;8(0):9-17. doi: <http://dx.doi.org/10.1016/j.epidem.2014.06.001>.

7. Omori R, Chemaitelly H, Abu-Raddad LJ. Dynamics of non-cohabiting sex partnering in sub-Saharan Africa: a modelling study with implications for HIV transmission. Sex Transm Infect. 2015. doi: 10.1136/sextrans-2014-051925. PubMed PMID: 25746040.

8. Awad SF, Cuadros DF, Abu-Raddad LJ. Generic patterns of HIV infection distribution in human populations. Under preparation.

9. Barrat A, Barthelemy M, Pastor-Satorras R, Vespignani A. The architecture of complex weighted networks. Proceedings of the National Academy of Sciences of the United States of America. 2004;101(11):3747-52. Epub 2004/03/10. doi: 10.1073/pnas.0400087101. PubMed PMID: 15007165; PubMed Central PMCID: PMC374315.

10. Boccaletti S, Latora V, Moreno Y, Chavez M, Hwang DU. Complex networks: Structure and dynamics. Physics Reports. 2006;424(4–5):175-308. doi: 10.1016/j.physrep.2005.10.009.

11. Watts DJ, Strogatz SH. Collective dynamics of 'small-world' networks. Nature. 1998;393(6684):440-2. Epub 1998/06/12. doi: 10.1038/30918. PubMed PMID: 9623998.

12. Barabási AL. Linked: how everything is connected to everything else and what it means for business, science and everyday life: London: First Plume Printing; 2003.

13. Vickerman P, Martin NK, Hickman M. Understanding the trends in HIV and hepatitis C prevalence amongst injecting drug users in different settings—implications for intervention impact. Drug and alcohol dependence. 2012;123(1):122-31.

14. Pinkerton SD. Probability of HIV transmission during acute infection in Rakai, Uganda. AIDS Behav. 2008;12(5):677-84. Epub 2007/12/08. doi: 10.1007/s10461-007-9329-1. PubMed PMID: 18064559.

15. Hollingsworth TD, Anderson RM, Fraser C. HIV-1 transmission, by stage of infection. J Infect Dis. 2008;198(5):687-93. Epub 2008/07/30. doi: 10.1086/590501. PubMed PMID: 18662132.

16. Abu-Raddad LJ, Longini IM, Jr. No HIV stage is dominant in driving the HIV epidemic in sub-Saharan Africa. AIDS. 2008;22(9):1055-61. Epub 2008/06/04. doi: 10.1097/QAD.0b013e3282f8af84

00002030-200805310-00007 [pii]. PubMed PMID: 18520349.

17. Wawer MJ, Gray RH, Sewankambo NK, Serwadda D, Li X, Laeyendecker O, et al. Rates of HIV-1 transmission per coital act, by stage of HIV-1 infection, in Rakai, Uganda. J Infect Dis. 2005;191(9):1403-9. PubMed PMID: 15809897.

18. UNAIDS. UNAIDS Reference Group on Estimates, Modelling and Projections. 2007.

19. UNAIDS/WHO. *AIDS epidemic update 2010: UNAIDS fact sheet* (available at<http://www.unaids.org/documents/20101123_FS_SSA_em_en.pdf>, accessed 23 July 2012). 2010.

20. UNAIDS/WHO. Epidemiological data, HIV estimates 1990-2009 (available at <http://www.unaids.org/en/dataanalysis/epidemiology/)>. 2010.

21. Vickerman P, Martin NK, Hickman M. Understanding the trends in HIV and hepatitis C prevalence amongst injecting drug users in different settings--implications for intervention impact. Drug and alcohol dependence. 2012;123(1-3):122-31. Epub 2011/12/06. doi: 10.1016/j.drugalcdep.2011.10.032. PubMed PMID: 22138540.

22. Osburn WO, Fisher BE, Dowd KA, Urban G, Liu L, Ray SC, et al. Spontaneous control of primary hepatitis C virus infection and immunity against persistent reinfection. Gastroenterology. 2010;138(1):315-24. doi: 10.1053/j.gastro.2009.09.017. PubMed PMID: 19782080; PubMed Central PMCID: PMC2889495.

23. Grebely J, Page K, Sacks-Davis R, van der Loeff MS, Rice TM, Bruneau J, et al. The effects of female sex, viral genotype, and IL28B genotype on spontaneous clearance of acute hepatitis C virus infection. Hepatology (Baltimore, Md). 2014;59(1):109-20. doi: 10.1002/hep.26639. PubMed PMID: 23908124; PubMed Central PMCID: PMC3972017.

24. Vickerman P, Hickman M, May M, Kretzschmar M, Wiessing L. Can hepatitis C virus prevalence be used as a measure of injection-related human immunodeficiency virus risk in populations of injecting drug users? An ecological analysis. Addiction. 2010;105(2):311-8. Epub 2009/11/20. doi: 10.1111/j.1360-0443.2009.02759.x. PubMed PMID: 19922515.

25. Cohen MS, Chen YQ, McCauley M, Gamble T, Hosseinipour MC, Kumarasamy N, et al. Prevention of HIV-1 infection with early antiretroviral therapy. The New England journal of medicine. 2011;365(6):493-505. doi: 10.1056/NEJMoa1105243. PubMed PMID: 21767103; PubMed Central PMCID: PMC3200068.

26. Donnell D, Baeten JM, Kiarie J, Thomas KK, Stevens W, Cohen CR, et al. Heterosexual HIV-1 transmission after initiation of antiretroviral therapy: a prospective cohort analysis. Lancet. 2010;375(9731):2092-8. doi: 10.1016/S0140-6736(10)60705-2. PubMed PMID: 20537376; PubMed Central PMCID: PMC2922041.

27. Group HIVMCTaPEW. HIV treatment as prevention: models, data, and questions--towards evidence-based decision-making. PLoS Med. 2012;9(7):e1001259. doi: 10.1371/journal.pmed.1001259. PubMed PMID: 22802739; PubMed Central PMCID: PMC3393655.
