# Supplementary material for: HCV prevalence can predict HIV epidemic potential among people who inject drugs: mathematical modeling analysis
Source: BMC Public Health. 2016 Dec 3;16:1216. doi: 10.1186/s12889-016-3887-y (PMC5135754; doi:10.1186/s12889-016-3887-y)
Supplement: Additional file 2: — Models assumptions in terms of parameter values. (DOCX 95 kb) [file 12889_2016_3887_MOESM2_ESM.docx]

**Table S1**

**Table S1.** Models assumptions in terms of parameter values

| **Parameter** | **Symbol** | **Value** | **Range of values^*^** | **Reference** |
| --- | --- | --- | --- | --- |
| **HIV Biological parameters** |  |  |  |  |
| HIV Infectiousness ratio for injecting/sexual transmission | $R$ | 10 | N/A | [[1](#_ENREF_1)] |
| Probability of transmission per shared injection in each HIV stage: |  |  |  |  |
| Acute | $p_{I_{j,1}\to S_{i}}^{HIV}$ | $R\times0.03604=0.360$ | N/A | Calculation using $R$ and reference [[2](#_ENREF_2)] |
| Latent | $p_{I_{j,2}\to S_{i}}^{HIV}$ | $R\times0.0008=0.008$ | N/A | Calculation using $R$ and reference [[2](#_ENREF_2)] |
| Advanced | $p_{I_{j,3}\to S_{i}}^{HIV}$ | $R\times0.0042=0.042$ | N/A | Calculation using $R$ and reference [[2](#_ENREF_2)] |
| Duration of each HIV stage: |  |  |  |  |
| Acute | $1/\omega_{1}$ | 49 days | N/A | [[3-8](#_ENREF_3)] |
| Latent | $1/\omega_{2}$ | 9 years | N/A | [[3-8](#_ENREF_3)] |
| Advanced | $1/\omega_{3}$ | 2 years | N/A | [[3-8](#_ENREF_3)] |
| **HCV Biological parameters** |  |  |  |  |
| Infectiousness ratio of HCV to HIV | $F$ | 7.8 | 1-15 | Model fitting |
| Probability of transmission per shared injection in each HCV stage: |  |  |  |  |
| Acute | $p_{Y_{j,1}\to S_{i}}^{HCV}$ |  | N/A | [[1](#_ENREF_1)] |
| Chronic | $p_{Y_{j,2}\to S_{i}}^{HCV}$ | $F\times P_{I_{j,2}}^{HIV}=0.062$ | N/A | Calculation using $F$ |
| Secondary acute | $p_{Y_{j,3}\to S_{i}}^{HCV}$ |  | N/A | [[9](#_ENREF_9)] |
| Duration of each HCV stage: |  |  |  |  |
| Acute | $1/\delta_{1}$ | 16.5 weeks | N/A | [[10](#_ENREF_10)] |
| Secondary acute | $1/\delta_{2}$ | 4.1 weeks | N/A | [[9](#_ENREF_9)] |
| Proportion of virus clearance among: |  |  |  |  |
| Primary HCV infections | $\gamma_{1}$ | 25% | N/A | [[10](#_ENREF_10)] |
| HCV reinfections | $\gamma_{2}$ | 83% | N/A | [[9](#_ENREF_9)] |
| **Behavioral and demographic parameters** |  |  |  |  |
| Death rate | $\mu$  | $1/70\text{ }years=0.014$ | N/A | [[11](#_ENREF_11)] |
| Duration of injection career | $\eta$ | 10 years | N/A | [[1](#_ENREF_1)] |
| Degree of assortative mixing | $e$ | 0.3 | 0-1 | [[1](#_ENREF_1)] |
| Scale parameter in the gamma distribution of the population across risk groups | $b$ | 0.5 | 0.4-0.6 | Model fitting |
| Shape parameter in the gamma distribution of the population across risk groups | $a$ | 0.28 | 0.05-0.45 | Model fitting |
| Exponent parameter in the power law function of the risk behavior distribution | $\alpha$ | 2.0 | 1.0-2.6 | Model fitting |
| Number of sharing acts per partnership | $n_{I_{j,\beta}\leftrightarrow S_{i}}$ | 50 | N/A | Representative value |

^*^ In the sensitivity analyses

**REFERENCES**

1. Vickerman P, Martin NK, Hickman M. Understanding the trends in HIV and hepatitis C prevalence amongst injecting drug users in different settings—implications for intervention impact. Drug and alcohol dependence. 2012;123(1):122-31.

2. Abu-Raddad LJ, Longini Jr IM. No HIV stage is dominant in driving the HIV epidemic in sub-Saharan Africa. Aids. 2008;22(9):1055-61.

3. Wawer MJ, Gray RH, Sewankambo NK, Serwadda D, Li X, Laeyendecker O, et al. Rates of HIV-1 transmission per coital act, by stage of HIV-1 infection, in Rakai, Uganda. J Infect Dis. 2005;191(9):1403-9. PubMed PMID: 15809897.

4. Pinkerton SD. Probability of HIV transmission during acute infection in Rakai, Uganda. AIDS Behav. 2008;12(5):677-84. Epub 2007/12/08. doi: 10.1007/s10461-007-9329-1. PubMed PMID: 18064559.

5. UNAIDS. UNAIDS Reference Group on Estimates, Modelling and Projections. 2007.

6. UNAIDS/WHO. AIDS epidemic update 2007. 2007.

7. UNAIDS/WHO. *AIDS epidemic update 2010: UNAIDS fact sheet* (available at<http://www.unaids.org/documents/20101123_FS_SSA_em_en.pdf>, accessed 23 July 2012). 2010.

8. UNAIDS/WHO. Epidemiological data, HIV estimates 1990-2009 (available at <http://www.unaids.org/en/dataanalysis/epidemiology/)>. 2010.

9. Bain VG, Kaita KD, Yoshida EM, Swain MG, Heathcote EJ, Neumann AU, et al. A phase 2 study to evaluate the antiviral activity, safety, and pharmacokinetics of recombinant human albumin-interferon alfa fusion protein in genotype 1 chronic hepatitis C patients. Journal of hepatology. 2006;44(4):671-8.

10. Grebely J, Feld JJ, Applegate T, Matthews GV, Hellard M, Sherker A, et al. Plasma interferon‐gamma‐inducible protein‐10 (IP‐10) levels during acute hepatitis C virus infection. Hepatology. 2013;57(6):2124-34.

11. Hickman IJ, Powell EE, Prins JB, Clouston AD, Ash S, Purdie DM, et al. In overweight patients with chronic hepatitis C, circulating insulin is associated with hepatic fibrosis: implications for therapy. Journal of hepatology. 2003;39(6):1042-8.
