# Supplementary figures and images for: HCV prevalence can predict HIV epidemic potential among people who inject drugs: mathematical modeling analysis
Source: BMC Public Health. 2016 Dec 3;16:1216. doi: 10.1186/s12889-016-3887-y (PMC5135754; doi:10.1186/s12889-016-3887-y)

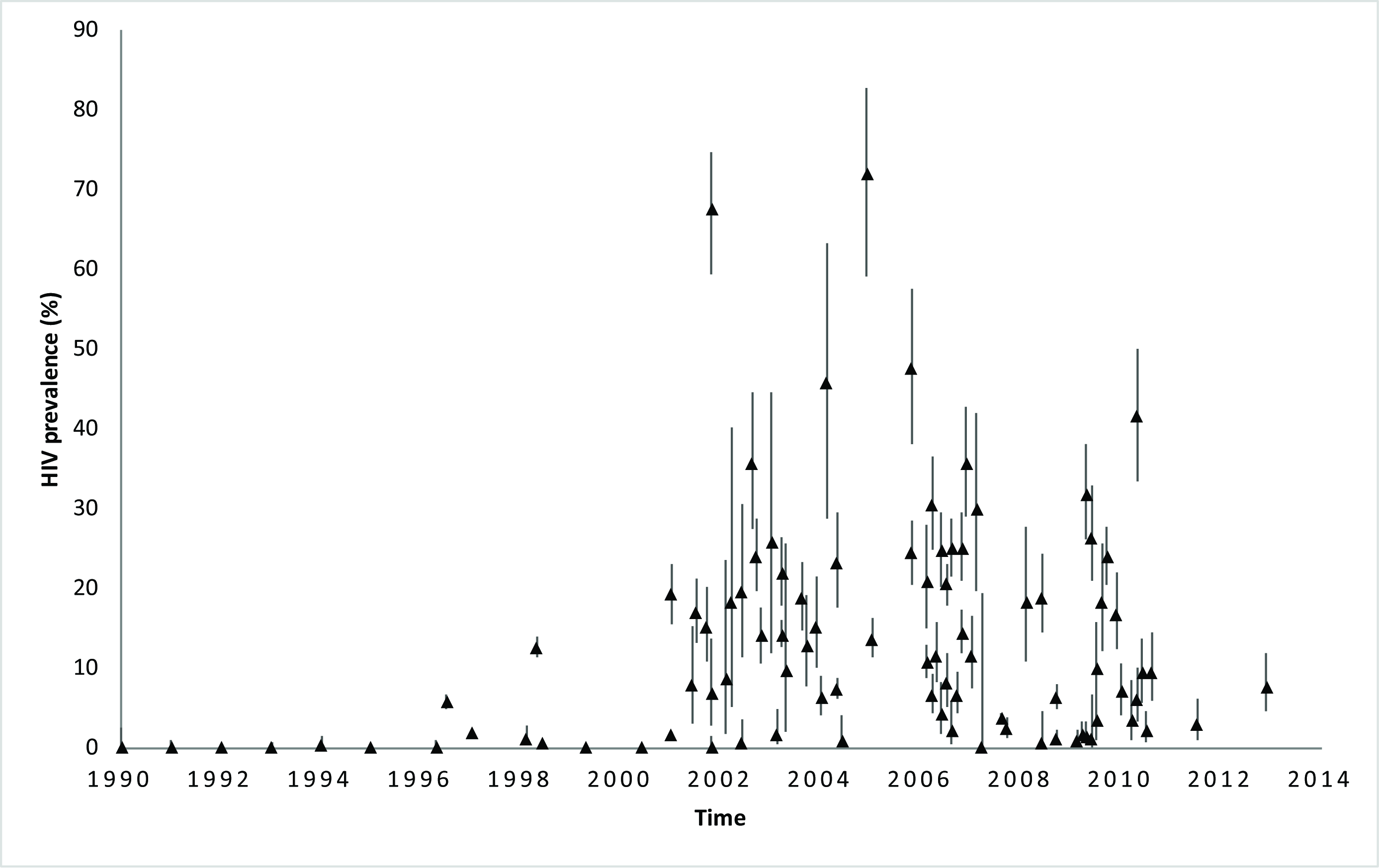

Supplement: Additional file 3: — Trend of HIV prevalence among PWID in Iran as described by available HIV point-prevalence measures 1990–2013. (TIF 1115 kb) [file 12889_2016_3887_MOESM3_ESM.tif]

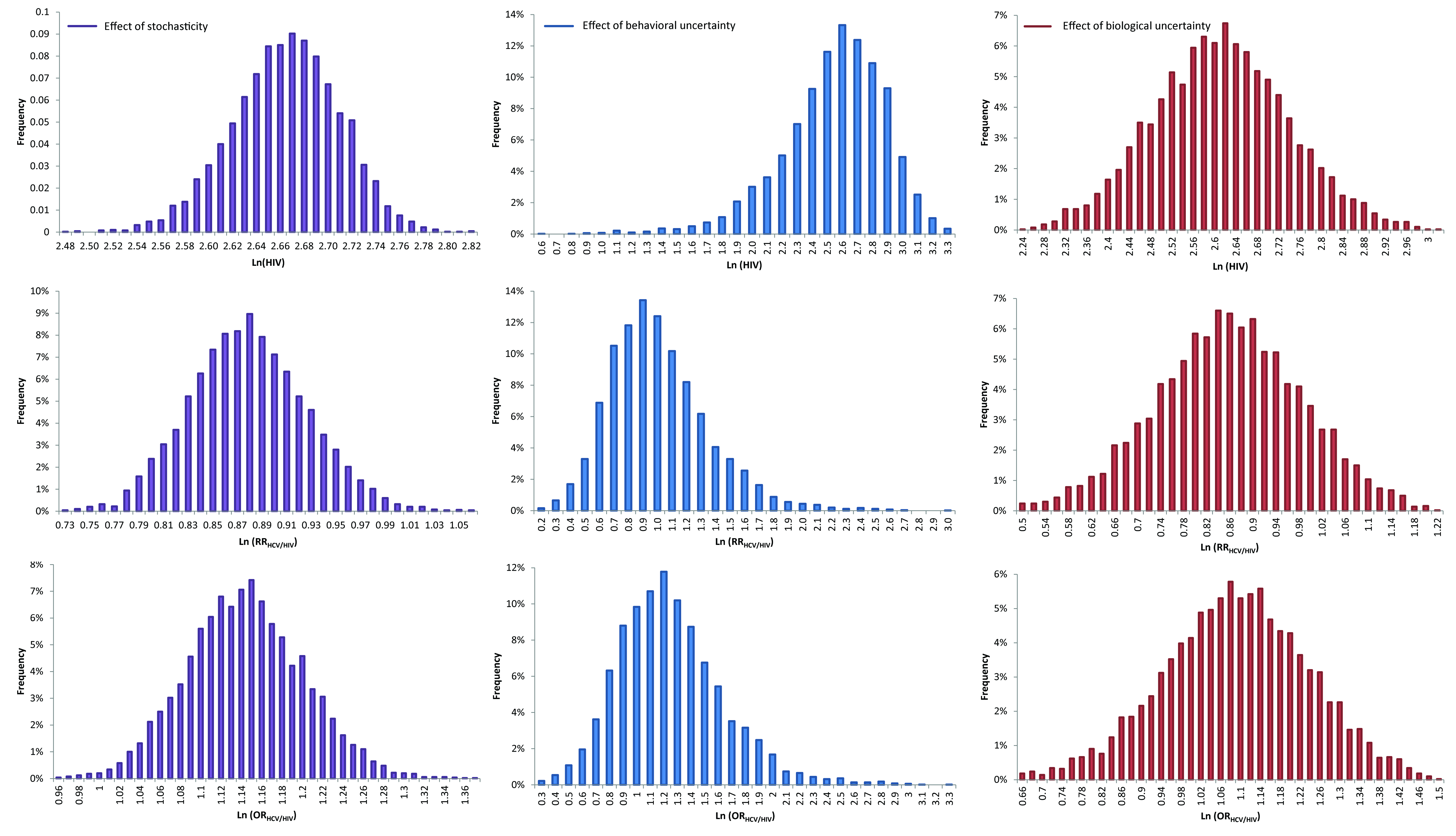

Supplement: Additional file 4: — Effect of stochasticity (purple) and of behavioral (blue) and biological (red) uncertainty on the modeling predictions of the endemic HIV prevalence, RR HCV/HIV and OR HCV/HIV at 59.4% HCV prevalence in Iran. (TIF 3208 kb) [file 12889_2016_3887_MOESM4_ESM.tif]
